# Vulnerability to Oxidative Stress *In Vitro* in Pathophysiology of Mitochondrial Short-Chain Acyl-CoA Dehydrogenase Deficiency: Response to Antioxidants

**DOI:** 10.1371/journal.pone.0017534

**Published:** 2011-04-01

**Authors:** Zarazuela Zolkipli, Christina B. Pedersen, Anne-Marie Lamhonwah, Niels Gregersen, Ingrid Tein

**Affiliations:** 1 Neurometabolic Research Laboratory, Division of Neurology, Department of Pediatrics, Hospital for Sick Children and Department of Laboratory Medicine and Pathobiology, University of Toronto, Toronto, Canada; 2 Research Unit for Molecular Medicine, Aarhus University Hospital, Skejby, Aarhus N, Denmark; McMaster University, Canada

## Abstract

**Objective:**

To elucidate the pathophysiology of SCAD deficient patients who have a unique neurological phenotype, among fatty acid oxidation disorders, with early developmental delay, CNS malformations, intractable seizures, myopathy and clinical signs suggesting oxidative stress.

**Methods:**

We studied skin fibroblast cultures from patients homozygous for ACADS common variant c.625G>A (n = 10), compound heterozygous for c.625G>A/c.319C>T (n = 3) or homozygous for pathogenic c.319C>T (n = 2) and c.1138C>T (n = 2) mutations compared to fibroblasts from patients with carnitine palmitoyltransferase 2 (CPT2) (n = 5), mitochondrial trifunctional protein (MTP)/long-chain L-3-hydroxyacyl-CoA dehydrogenase (LCHAD) (n = 7), and medium-chain acyl-CoA dehydrogenase (MCAD) deficiencies (n = 4) and normal controls (n = 9). All were exposed to 50 µM menadione at 37°C. Additonal conditions included exposure to 39°C and/or hypoglycemia. Time to 100% cell death was confirmed with trypan blue dye exclusion. Experiments were repeated with antioxidants (Vitamins C and E or N-acetylcysteine), Bezafibrate or glucose and temperature rescue.

**Results:**

The most significant risk factor for vulnerability to menadione-induced oxidative stress was the presence of a FAO defect. SCADD fibroblasts were the most vulnerable compared to other FAO disorders and controls, and were similarly affected, independent of genotype. Cell death was exacerbated by hyperthermia and/or hypoglycemia. Hyperthermia was a more significant independent risk factor than hypoglycemia. Rescue significantly prolonged survival. Incubation with antioxidants and Bezafibrate significantly increased viability of SCADD fibroblasts.

**Interpretation:**

Vulnerability to oxidative stress likely contributes to neurotoxicity of SCADD regardless of *ACADS* genotype and is significantly exacerbated by hyperthermia. We recommend rigorous temperature control in SCADD patients during acute illness. Antioxidants and Bezafibrate may also prove instrumental in their management.

## Introduction

Cellular energy metabolism is largely sustained by mitochondrial β-oxidation of fatty acids when carbohydrate stores are depleted after fasting or prolonged exercise. Whereas the clinical presentations of long- and medium-chain fatty acid oxidation disorders (FAODs) are primarily of hepatic or cardiac dysfunction, short-chain acyl-CoA dehydrogenase deficiency (SCADD) (OMIM # 201470) is predominantly neurological in presentation with developmental delay, seizures, hypotonia and myopathy [1], [2]. Thirty-five SCAD gene (*ACADS*) inactivating mutations have been described in this autosomal recessive disorder [1]–[3]. The molecular phenomenon of the common *ACADS* variations, c.625G>A and c.511C>T in SCADD is unique. These variations are identified in SCADD patients, but also in various general populations at allelic frequencies of 22%–43% and 3%–8% respectively [4]–[7]. The biochemical consequences are SCAD protein misfolding and aggregation described in *in vitro* mitochondrial import studies [8], which correlate poorly with clinical severity. Patients with the *ACADS* gene spectrum may remain asymptomatic or have muscle and neuronal toxicity. The speculated role of additional gene and/or environmental modifiers needs to be better understood.

We studied a girl with progressive limb-girdle myopathy, ptosis, facial weakness and progressive external ophthalmoplegia and features overlapping with mitochondrial Complex I deficiency including cataracts and cardiomyopathy, confirmed to have SCADD with homozygosity for c.319 C>T mutation [9]. As a result of her myopathy, she became wheelchair-dependent by 5 years of age. Independent 1 year trials of a high carbohydrate/low fat diet, L-carnitine, and riboflavin did not improve her strength. Her total plasma aldehydes by GC/MS were significantly elevated at 2997.6 nM, (controls 2041.3±228.3, n = 12) [10]. A comparable rise was reported in complex I deficiency (3377.8±595.7, n = 14). We thus instituted a trial of Vitamins C 200 mg twice a day and E 400 IU daily. After 6 months, she had a 6-fold increase in endurance time on sustained deltoid abduction.

This supported our speculation that increased oxidative stress is a pathogenetic mechanism in SCADD. Mitochondria are a major site of reactive oxygen species (ROS) production, which are pathologic when excessive. Mitochondrial dysfunction with increased ROS secondary to SCADD could explain the overlapping clinical features of complex I and SCADD as seen in our patient. The possible association between SCADD and oxidative stress in vitro has been investigated. EMA inhibits mitochondrial creatine kinase activity [11], [12], increases lipid and protein oxidation products, and decreases glutathione (GSH) levels in rat cerebral cortex [13]. In human skeletal muscle, EMA inhibits electron transport at complexes I–III and II–III [14]. Given that the respiratory chain (RC) may generate excessive ROS from defective electron transport [15], at complex I and Complex III [16], [17], we speculate that EMA accumulation may contribute to oxidative stress and the neurological symptoms in SCADD patients by this mechanism. Furthermore, ROS reportedly affects the permeability of the blood brain barrier, enhancing neuronal vulnerability to ROS toxicity [18].

The second possible mechanism is a direct consequence of the SCAD protein misfolding. The mitochondrial RC produces superoxides or hydroxyl radicals (OH^−^) from the interaction of molecular oxygen with semi-quinone or -flavone species [19], [20]. SCAD is an FAD-linked dehydrogenase, and a defect in SCAD function, due to misfolding and defective interaction with functional partners e.g. electron transfer flavoprotein (ETF), may thus lead to the production of superoxides in proximity to sites of semi-flavone production.

A third potential mechanism relates to cellular antioxidant status. Oxidative stress is due to an imbalance between excessive ROS generation and antioxidant capacity, such as superoxide dismutase (SOD) and glutathione peroxidase activity [20]–[22]. The two intracellular SOD enzymes are intramitochondrial manganese superoxide dismutase (SOD2) and cytosolic copper zinc SOD (SOD1). A recent proteomic analysis of mitochondria from c.625G>A homozygous patient fibroblasts (n = 4) revealed reduced SOD2 protein and mRNA expression compared to controls (n = 6), rendering them more vulnerable to oxidative stress [23]. Sequencing of the SOD2 gene did not demonstrate abnormalities, suggesting possible intrinsic dysregulation of SOD2.

Therefore oxidative stress in SCADD could be the result of associated biochemical abnormalities combined with intrinsic antioxidant dysfunction and exogenous stressors. We plan to first identify the selective vulnerability to oxidative stress, *in vitro*, in SCADD patient fibroblasts by exposure to menadione, a free radical amplifier, both with and without stressors. Menadione generates ROS through redox cycling, and has been shown to decrease mitochondrial membrane potential and trigger cytochrome c redistribution to the cytosol [22], [24], [25]. Multiple redundant cell death pathways are activated by menadione, and poly ADP ribose polymerase (PARP) plays an essential role in mediating each of them [25]. As stressors, we have chosen hyperthermia at 39°C, which increases SCAD protein aggregation [8], and hypoglycemia, which limits the cellular bioenergetic source and puts further stress on an already dysfunctional FAO pathway. Both of these stressors are common well-recognized exogenous triggers for catabolic crisis in FAO disorders. We will then test the therapeutic efficacy of specific pathophysiology-based treatment interventions.

We recognize that the extrapolation of cellular pathophysiologic mechanisms from fibroblasts to neuronal tissue is suboptimal, however this is generally the primary and often only patient cell type available to study *in vitro*. We plan to compare the results from SCAD deficient fibroblasts to those with medium and long-chain ACAD deficiencies. The family of ACAD deficiencies have similarities such as intermittent metabolic crises with hypoketotic hypoglycemia and clinical variability. The long-chain ACAD deficiencies present with hepatic, cardiac and skeletal muscle symptoms and signs. Medium-chain ACAD deficiencies present with hepatic features, whereas SCAD deficiency is primarily neurological/neuromuscular in presentation. Based on our index case, we plan to study the relative effect of oxidative stress in the short-, medium-, and long-chain ACAD deficiencies at a cellular level in order to determine whether this is a selective mechanistic pathway that contributes to the chronic neurotoxicity in SCADD.

## Materials and Methods

### Ethics Statement

The Toronto Hospital for Sick Children Research Ethics Board and the Danish Ethical Committee (M-20070150) approved the use of anonymized patient fibroblasts for this study and waived the need for informed consent based on a three-tier de-identification which totally precluded any possibility of patient identification.

### Patients

De-identified skin fibroblast cultures from clinically affected pediatric patients (<18 years of age) with SCAD (n = 17), MCAD (n = 4), CPT2 (n = 5), MTP (n = 6) and LCHAD (n = 1) enzyme deficiencies were provided by the Research Unit for Molecular Medicine, Aarhus, Denmark and the Test Development Laboratory, Children's Medical Center Dallas, Texas. The SCAD deficient patient fibroblasts had the following genotypes; *ACADS* c.1138T/1138T (n = 2), c.319 T/319T (n = 2), c.625A/319T (n = 3), and c.625A/625A (n = 10). The MCAD deficient patient fibroblasts had the following genotypes: *ACADM* c.985A>G/PTC (premature stop codon), PTC/PTC, STOP (stop codon mutation)/STOP, and missense/missense mutations. Four of the MTP patients had the following genotypes: *HADHA* c.1528G>C/PTC, *HADHA* c.1528G>C/missense, *HADHB* splice mutation/splice mutation, and *HADHB* missense/PTC.

### Controls

De-identified skin fibroblasts from asymptomatic individuals within the pediatric age group were provided by the Tissue Culture Laboratory, Toronto Hospital for Sick Children and the Research Unit for Molecular Medicine, Denmark, who sequenced for the *ACADS* c.625G>A genotype in all control fibroblasts which included: c.[625G]+[625G] (G/G) (n = 5), c.[625G]+[625A] (G/A) (n = 4) and c.[625A]+[625A] (A/A) (n = 4). All controls were homozygous for the wild-type sequence at nucleotide position 511 (c.511C).

All fibroblasts tested negative for mycoplasma.

### Menadione toxicity assay

Skin fibroblasts were seeded in 24-well plates (Biosciences) with 20,000 cells per well. Fibroblasts were grown to a monolayer of confluence in α-MEM containing 2.0 g/L of glucose and 10% FCS at 37°C, in 5% CO_2_ then washed and incubated with 50 µmol/L menadione in serum-free α-MEM. Cells were incubated in media with or without glucose, and at 37°C or at 39°C. Cell viability was evaluated by light microscopy (LM) every hour until 100% cell death in the SCAD deficient cells, and every four hours until approximately 50% cell death, then every hour until 100% cell death in the control cells. Time to 100% cell death (in hours) was confirmed with 0.4% trypan-blue dye exclusion (Gibco) by LM. If on examination of trypan-blue dye exclusion, evaluation of cell death was <100%, the experiment was repeated until 100% death was confirmed. In order to confirm reproducibility, each experiment was performed in triplicate, on at least 8 separate occasions. The mean of these technical replicates were used in the final statistical analysis.

### Rescue

Rescue of hypoglycemia with α-MEM+glucose, and rescue of hyperthermia by reducing incubator temperature from 39°C to 37°C was performed at <40% cell death in 11 SCADD fibroblast lines (c.625A/625A (n = 5), c.319T/625A (n = 3), c.319T/319T (n = 2) and c.1138T/1138T (n = 1)) after menadione exposure. Time to 100% cell death was subsequently confirmed as above.

### Intervention

Fibroblasts were incubated with menadione and i) antioxidant vitamin C 0.8 mg/dL and vitamin E 0.9 mg/dL (antiO) (Sigma-Aldrich), ii) N-acetyl-cysteine (NAC) 0.5 or 5 mM (Sigma-Aldrich), preceded by 24 hours of pre-incubation with NAC prior to menadione exposure or iii) Bezafibrate (B+) (Cayman Chemicals) 200 and 400 µmol/L in the SCADD fibroblasts only, preceded by 48 hours of pre-incubation with Bezafibrate.

### Statistical analysis

Results are expressed as mean ± SEM (hours). All values underwent logarithmic transformation with subsequent confirmation of normality using the D'Agostino Pearson normality test, prior to statistical analysis with one-way ANOVA and Bonferroni's sub-group analysis (GraphPad Prism software). Logarithmic transformation was performed due to unequal sample sizes between groups. P-values <0.05 were considered significant.

## Results

### Menadione toxicity assay

**Figure 1:**
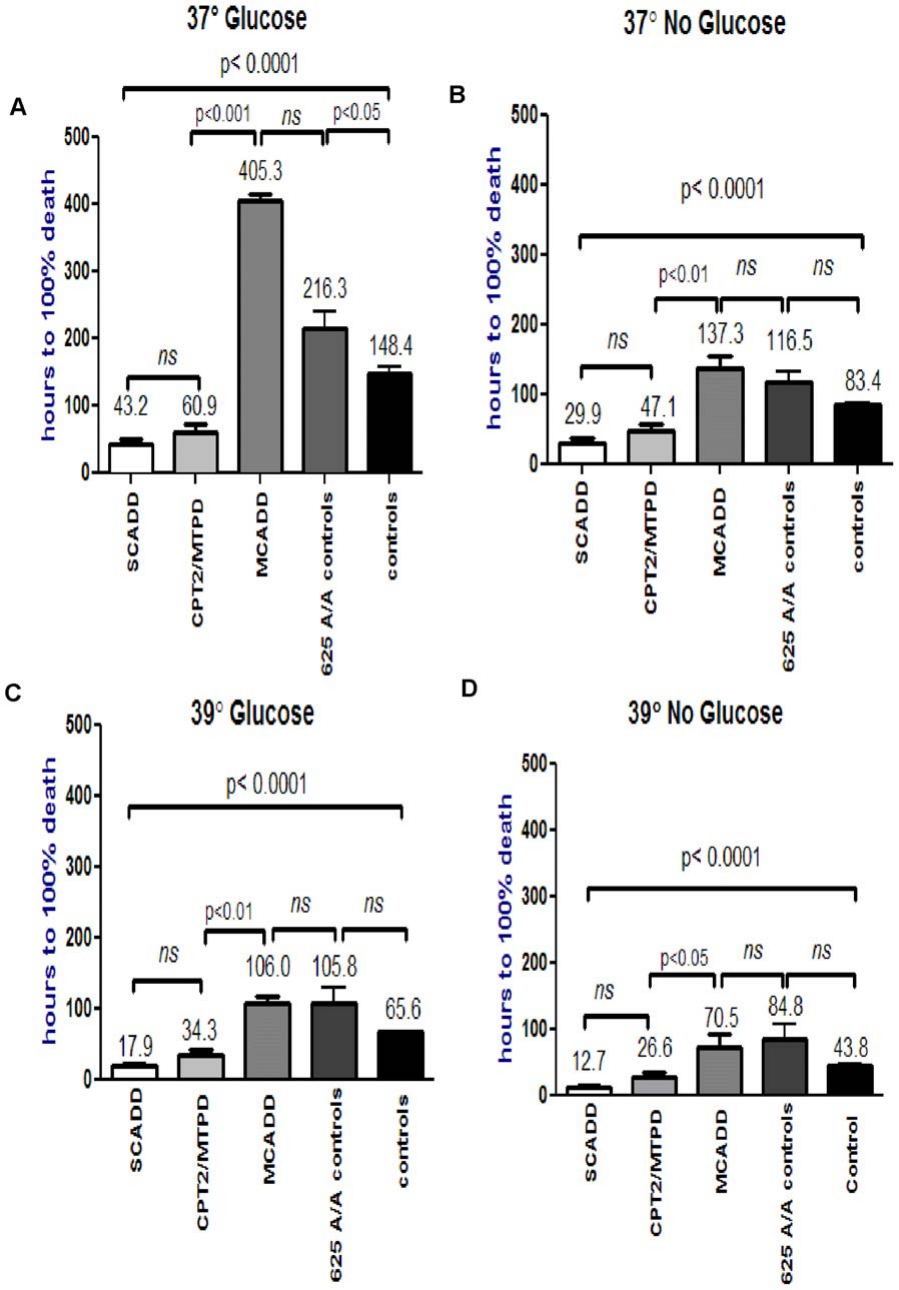
Comparison of menadione toxicity in short-chain acyl-CoA dehydrogenase deficiency (SCADD), medium-chain acyl-CoA dehydrogenase deficiency (MCADD), carnitine palmitoyltransferase 2 deficiency (CPT2D) and mitochondrial trifunctional protein deficiency (MTPD)/long-chain L-3hydroxyacyl-CoA dehydrogenase deficiency patients and 625A/A controls (n = 4) and 625G/A and G/G (n = 9) control data under the 4 experimental conditions. One-way ANOVA and Bonferroni's Mutiple Comparison test compares menadione toxicity, after logarithmic transformation to ensure Gaussian distribution. The ANOVA shows significant differences between all groups, in all 4 experimental conditions, indicated by the error bar spanning all cell lines, p<0.0001(Table S1). The Bonferroni subgroup analyses are summarized in Table S3, and indicated by the error bars comparing 2 cell lines. Figures 1a to d displays the significantly reduced time to 100% cell death in the SCADD and CPT2/MTPD patient cell lines, compared to the 625A/A control and normal control (625 G/G and 625 G/A) cell lines. The MCADD cell lines survived significantly longer than the normal control cell lines, only at physiological conditions (Table S3). Figures 1b to 1d show the progressive reduction in survival time to 100% cell death in all cell lines, with the addition of glucose deprivation (Figure 1b), elevated temperature (Figure 1c), or both (Figure 1d).

Fibroblast skin cultures from 17 patients with SCADD, 12 with long-chain FAODs including CPT2 (n = 5) and MTP/LCHAD (n = 7) deficiencies, 4 with MCADD, 4 reportedly asymptomatic c.625G>A homozygotes (A/A), and 9 normal controls with genotypes of c.625G/625G (G/G, n = 5) and c.625G/625A (G/A, n = 4) were studied. In SCADD, no consistent genotype-phenotype correlations have been identified [8], therefore all 17 patient cell lines of varying genotypes were included in one group. Each cell line underwent menadione exposure with or without glucose (hypoglycemia), and at 37°C or 39°C (hyperthermia) (Fig. 1A to D, Table S1). The most striking result was the exquisite vulnerability of the SCADD patient fibroblasts compared to controls under each of the 4 conditions, p<0.001 (Table S3). The long-chain FAOD fibroblasts (CPT2/MTP) were also significantly more vulnerable compared to controls under each of the four conditions, p<0.05. Menadione toxicity in the CPT2/MTP patient fibroblasts was comparable to that in the SCADD patient fibroblasts, p>0.05 (Table S3). The time to 100% death in the MCADD cell lines was longer than in the control cells (625G/G and 625 G/A) only under physiological conditions, p<0.05, but was not significantly different when glucose deprivation and/or hyperthermia were added, p>0.05. In addition, MCADD fibroblasts were not significantly different from the 625 A/A controls under all four experimental conditions, p>0.05 (Table S3). In comparison of the controls, the 625G/G and 625G/A control fibroblasts (n = 9) were unexpectedly more vulnerable than the 625A/A control fibroblasts (n = 4) (Fig. 1A to D), also reaching significance only under physiological conditions, p<0.05 (Table S3). This is concordant with results of mitochondrial proteomics analysis which indicated higher levels of SOD2 expression in 625A/A control fibroblasts compared to lower SOD2 expression in 625G/G and 625G/A control fibroblasts [23].

In comparison of the individual effects of hypoglycemia or hyperthermia on viability in the SCADD patient fibroblasts, hyperthermia had the more significant effect, p<0.01, compared to hypoglycemia alone, p = 0.05 (Table S2). Although adverse conditions exacerbated vulnerability in all patient fibroblasts, the overall net effect was the most significant for the SCADD fibroblasts in all 4 experimental conditions, as shown by the short survival times, compared to controls, p<0.001 (Table S3).

**Figure 2:**
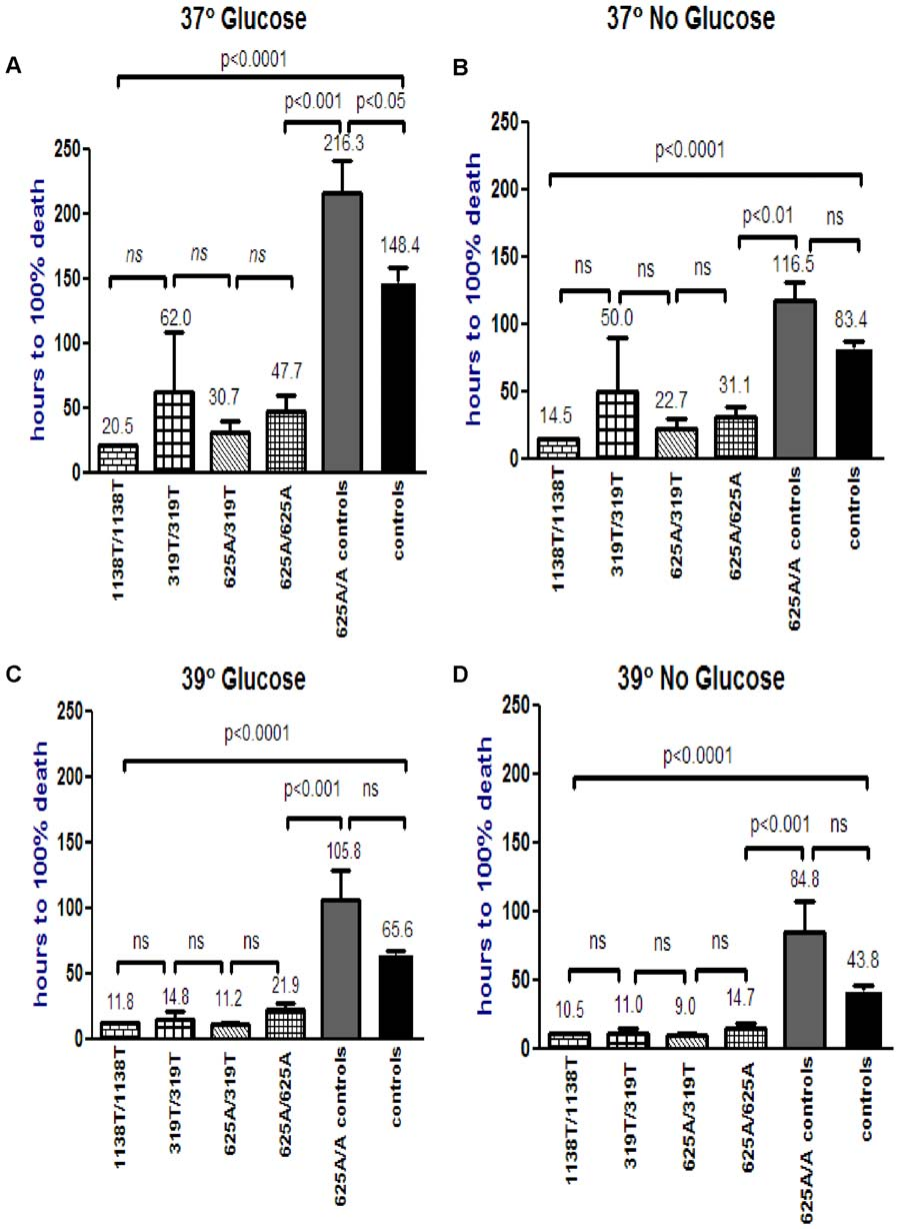
Menadione toxicity in different SCAD genotypes and controls under variable conditions. Patient fibroblasts with *ACADS* 1138T/T homozygous (n = 2), 319T/T homozygous (n = 2) and 625A/319T heterozygous (n = 3) mutations and 625A/A symptomatic homozygotes (n = 10) are compared to 625A/A (n = 4) and 625G/A and G/G controls (n = 9) by ANOVA, as indicated by the error bar spanning all cell lines, p<0.0001. Figures 2a to d shows that there were no significant differences in the time to 100% cell death between the 1138T/T, 319T/T, 625A/319T and 625A/A SCADD patient cell lines. However, the patient cell line survival times were significantly reduced when compared to the 625A/A control and normal 625G/A and G/G control cell lines, except for 319T/T, which was significantly different only at 39°C (Table S4).

Figure 2 shows the results of the SCADD fibroblasts divided into the four *ACADS* genotypes. There was no significant difference between the c.625G>A homozygotes (n = 10), c.625G>A/c.319C>T compound heterozygotes (n = 3) and pathogenic c.319C>T (n = 2) and c.1138C>T (n = 2) homozygotes, but there was a significant difference between each of the SCADD patient groups when compared to the c.625G>A controls, in all experimental conditions, except for c.319C>T (n = 2) which reached significance only under 39°C with and without glucose (Fig. 2A to D, Table S4). It is intriguing that the symptomatic common variant c.625G>A homozygous fibroblasts, most of which have been shown to have a considerable residual SCAD enzyme activity of approximately 50% of control [23], were just as susceptible as the fibroblasts with pathogenic mutations, which have residual SCAD enzyme activities of ∼5%. This further supports the speculation that although the biochemical abnormalities in SCADD may in part contribute to the pathophysiology, there are other modifiers contributing to the pathophysiology of the *ACADS* gene spectrum [5].

### Rescue in SCADD fibroblasts

To simulate resuscitation in an acute catabolic crisis, SCADD patient fibroblasts of all 4 genotypes (n = 11) were rescued prior to 40% cell death (Figure S1). (i) After exposure to menadione and hypoglycemia at 37°C (column 2), SCADD cells were rescued with glucose which resulted in increased survival (column 3). This approximated the survival of cells exposed to menadione at physiological conditions (column 1), p = 0.2. (ii) After exposure to menadione at 39°C, cells were rescued by reducing the incubator temperature to 37°C (column 5), p<0.0001. Survival also approximated baseline (column 1). However, the net increase in survival was 3.1-fold, p<0.0001, compared to glucose rescue of 1.5-fold, p<0.0001. (iii) After exposure to menadione with hypoglycemia and hyperthermia, simultaneous glucose and temperature rescue (column 8) had a more significant effect (2.9-fold), p<0.0001, than glucose rescue alone at 39°C (1.3-fold), p<0.0003. These results are concordant with the earlier finding that hyperthermia more significantly affects SCADD cell viability than hypoglycemia alone. It is striking that rescue restored survival to the original baseline, despite the additional exposure of an adverse factor. We can conclude that rescue at an early stage is effective.

### Interventions

Given that oxidative stress can be reduced with antioxidants, we evaluated the effects of vitamin C 0.8 mg/dL and vitamin E 0.9 mg/dL (AO), and N-acetyl-cysteine (NAC) (0.5 and 5 mM) in the SCADD fibroblasts. We also evaluated the effect of Bezafibrate (B+) (200 and 400 µmol/L) which is a pan-peroxisome proliferator activated receptor (PPAR) agonist reported to restore deficient FAO rates in CPT2 and VLCAD fibroblasts [26]. We initially evaluated the effects of Bezafibrate on the long-chain FAOD group, with the expectation that restoration of FAO would reduce oxidative stress. Bezafibrate has so far not been investigated in SCADD, however, if short-chain FAO were also restored by Bezafibrate, viability in the SCADD fibroblasts might similarly be enhanced.

**Figure 3:**
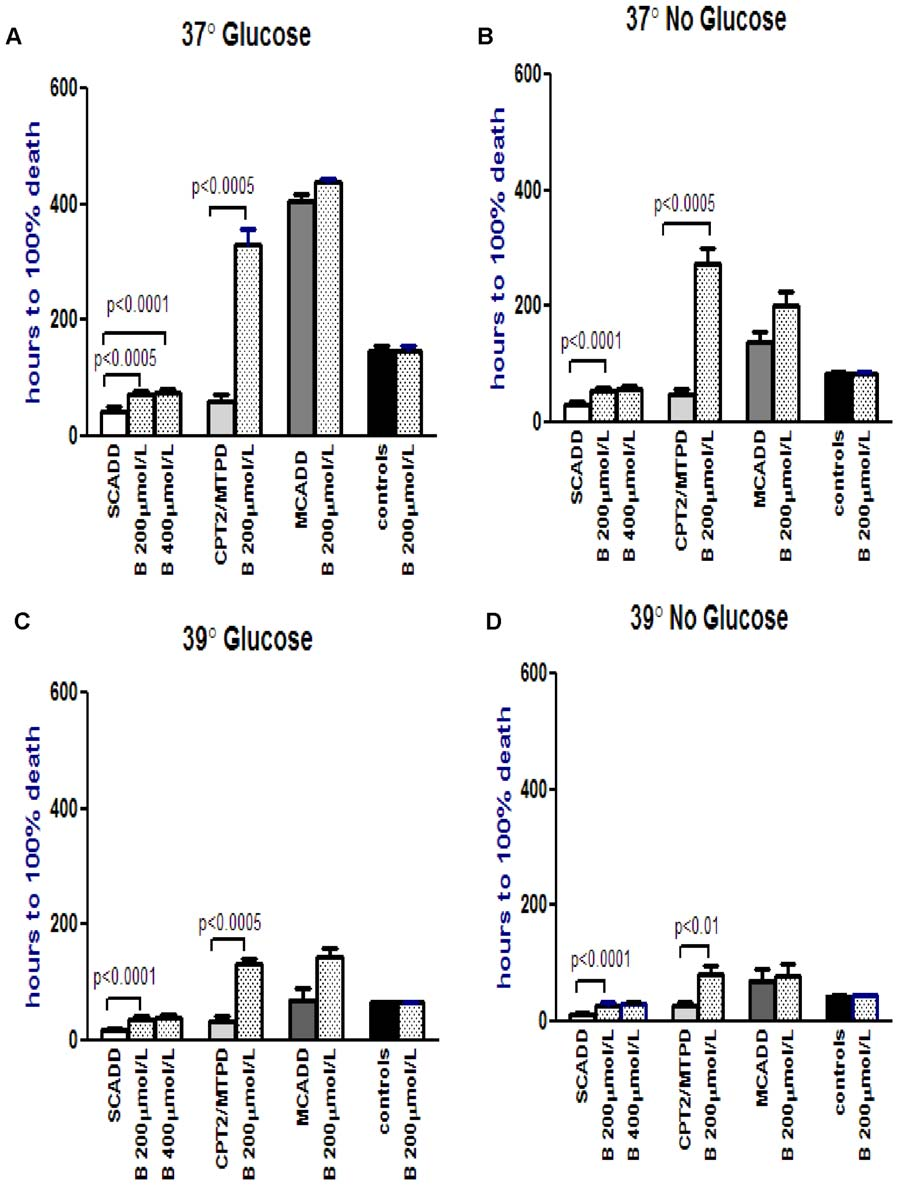
Effect of Bezafibrate (B) intervention (200 µmol/L, and 400 µmol/L in SCADD fibroblasts only) on menadione toxicity in each FAO disorder under variable conditions. Logarithmic transformation followed by paired t-test compares menadione toxicity in each FAOD with and without Bezafibrate, under each experimental condition. This figure shows that Bezafibrate significantly reduced menadione toxicity in the SCAD and MTP/CPT2 deficient patient fibroblasts only, under all 4 experimental conditions (Table S5).

**Figure 4:**
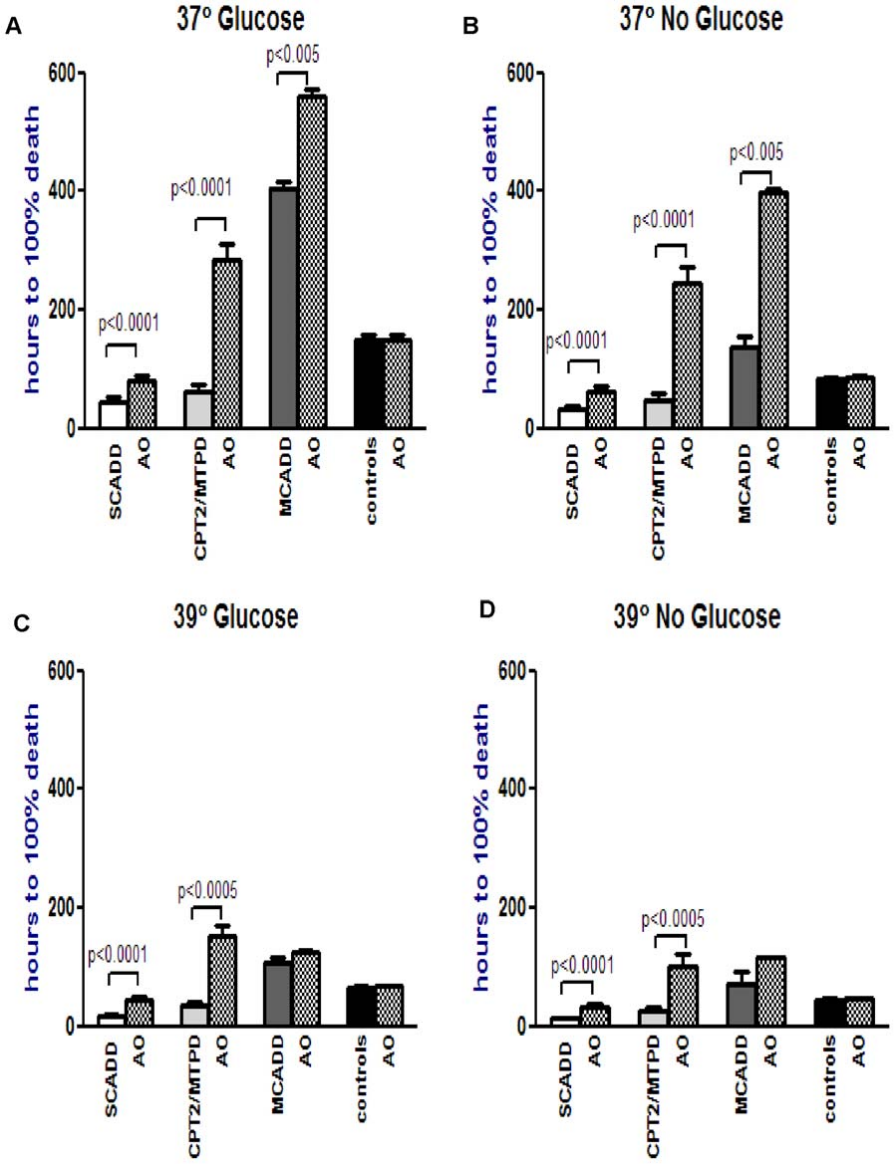
Effect of Antioxidant (AO) intervention on menadione toxicity in each FAO disorder under variable conditions. Logarithmic transformation followed by paired t-test compares menadione toxicity in each FAOD with and without antioxidants, under each experimental condition. This figure shows that antioxidants significantly increased survival time in the SCADD and CPT2/MTPD patient fibroblasts under all 4 conditions, and in the MCADD fibroblasts only at 37° with/without glucose (Table S6).

In the Bezafibrate and AO groups (Figure 3 and 4 respectively), there was significant improvement in viability in the SCADD (up to 2.3×, p<0.0005 and 2.5×, p<0.0001 respectively) and CPT2/MTP deficient (up to 5.8×, p<0.01 and 5.2×, p<0.0005) groups under all four conditions (Fig 3 A–D and 4 A–D, Tables S5, S6). There was no significant difference when Bezafibrate exposure was increased to 400 µmol/L in the SCADD cells, p>0.05. MCADD and control fibroblasts did not show any significant response to Bezafibrate, p>0.05 (Table S5). The MCADD cells did show significant response to AO at 37°C with and without glucose (Table S6).

**Figure 5:**
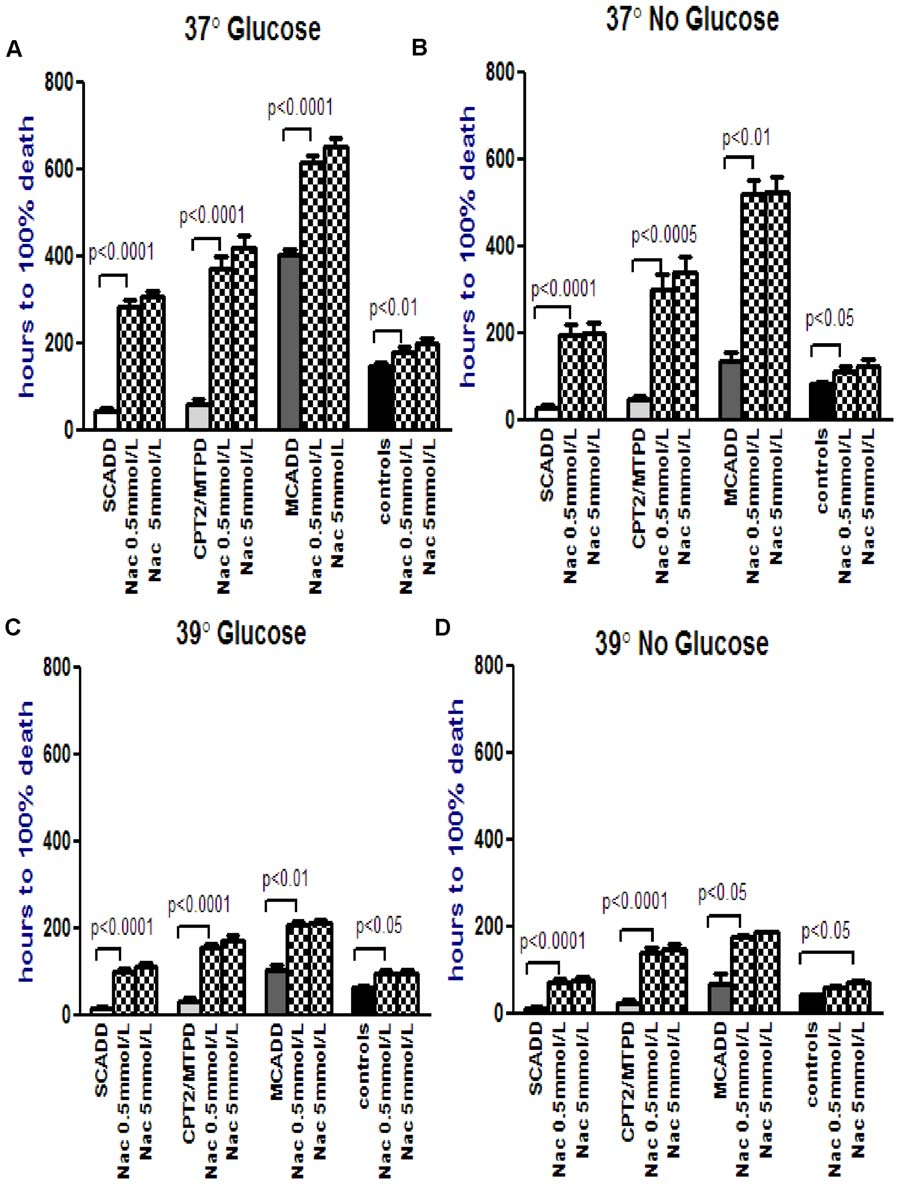
Effect of N-acetyl-cysteine (NAC, 0.5 and 5 mmol/L) intervention on menadione toxicity in each FAO disorder under variable conditions. Logarithmic transformation followed by paired t-test compares menadione toxicity in each FAOD with and without NAC, under each experimental condition. This figure shows that NAC at both concentrations was significantly effective for SCADD, CPT2/MTPD, MCADD patient fibroblasts under all experimental conditions. In the control fibroblasts, NAC at 5 mmol/L was effective under all experimental conditions.

In the NAC group (Fig. 5), the response to treatment was even more significant in the SCADD (up to 7.1×, p<0.0001) and CPT2/MTP deficient (up to 7.2×, p<0.0005) groups (Fig. 5A–D, Table S7). The MCADD fibroblasts also showed significant effects with NAC under all 4 experimental conditions, p<0.05. In the patient cells, there was no significant difference in effect between NAC concentrations of 0.5 mM or 5 mM, p>0.05. The control fibroblasts showed significant response under all 4 experimental conditions to NAC 5 mmol/L, p<0.05 (Table S7).

In summary, the SCADD and CPT2/MTP deficient fibroblasts showed a significant increase in viability with all 3 treatments. NAC had the most significant effect. The SCADD and CPT2/MTP fibroblasts were the most vulnerable to menadione- induced oxidative stress, suggesting that these cell lines had higher levels of cellular ROS. Therefore, the anti-oxidant intervention was most significant in these groups. The response to Bezafibrate in SCADD fibroblasts suggests there may be some restoration of short-chain FAO by PPAR regulation of the *ACADS* gene, thereby reducing the biochemical abnormalities arising from the SCAD block and the resultant vulnerability to oxidative stress.

## Discussion

We pursued the hypothesis that SCADD patients may have increased vulnerability to oxidative stress, based on the clinical and biochemical features of our index case [9], [10], the published mechanisms for EMA-induced oxidative stress [11]–[14], the cellular consequences of the SCAD protein misfolding [8], and our recent demonstration of antioxidant dysfunction in SCADD fibroblasts [23]. The relationship between abnormal FAO and secondary mitochondrial dysfunction is likely multifactorial. Our studies provide evidence of increased vulnerability to oxidative stress in SCADD fibroblasts, with exacerbation by hyperthermia and hypoglycemia. This exacerbation is of clinical relevance given that individuals with *ACADS* gene spectrum e.g those identified on newborn screening may remain asymptomatic until exposed to a triggering illness. This exacerbation is also concordant with mitochondrial import studies showing increased aggregation of SCAD proteins with hyperthermia [8]. Given that both protein aggregation and ROS production are aggravated by hyperthermia, this further highlights the interrelationship between these two mechanisms. The resulting oxidative imbalance from increased ROS production and from antioxidant dysfunction [23] may thereby exceed a subclinical threshold in affected individuals, giving rise to clinical disease. The results of our studies are therefore concordant with our hypothesis of increased vulnerability to oxidative stress in the pathogenetic mechanism of SCADD. These results also suggest an interweaving of pathogenetic mechanisms in SCADD namely increased oxidative stress, with the previous studies which demonstrated antioxidant dysfunction [23] and protein misfolding [8]. Furthermore, given that the SCAD protein is nuclear-encoded, the demonstrated abnormalities in cultured skin fibroblasts can be extrapolated to other cell types in the central and peripheral nervous system and likely contribute to the neurological and neuromuscular phenotypes of SCAD deficiency.

It is possible that significant oxidative imbalance is required prior to the manifestation of clinical symptoms. This may explain the discrepancy between the frequency of common SCAD variations in the general population, and the prevalence of clinically manifest SCADD. The vulnerability to oxidative stress appears to be independent of the *ACADS* genotype, concordant with previous reports of inconsistent genotype-phenotype correlations [1], [27]. A further example is the contrasting clinical phenotypes of c.625G>A homozygosity which may be explained by our recent description of decreased SOD2 expression in the affected patient vs ‘asymptomatic control’ fibroblasts [23]. In addition, exogenous stressors, such as hypoglycemia and fever, may influence the clinical phenotype. Oxidative stress with hydrogen peroxide, under heat stress, has been shown to impair the heat stress response (HSP40/HSP70), delay unfolded protein recovery and enhance loss of mitochondrial membrane potential [28].

In mitochondrial Complex I deficiency with the cardiomyopathy and cataracts phenotype, it has been proposed that significant induction of SOD2 may result from a temporarily much elevated superoxide production rate in the presence of an abnormally reduced redox state, as occurs in anoxia reperfusion injury [29]. Superoxide specifically attacks (4Fe-4S) centres in Complex I and II resulting in release of free iron in mitochondria and cytosol [30], [31], which generates excessive OH^−^ [32]. This could be the mechanism in our SCADD fibroblasts, with exacerbation by heat stress due to protein unfolding [8].

Oxidative stress is likely to trigger pro-apoptotic signaling cascades [33]. Of the 7 glutathione peroxidases in mammals, GPx4 is specific for phospholipid hydroperoxides in membranes, and is shown to be significant for neuronal survival [33]. GPx4 senses and translates oxidative stress into a 12/15-lipoxygenase dependent- and apoptosis-inducing factor-mediated cell death pathway. In GPx4-knockout cells, lipid peroxidative injury was the key mediator of cell death and was efficiently prevented by Vitamin E. Vitamins E and C have been reported to neutralize free radicals [34]. We evaluated these effects at physiological plasma concentrations. NAC has ROS scavenging actions, but is also a precursor for glutathione synthesis, and thus essential for the effects of GPx [35].

Intervention with Bezafibrate resulted in significant improvement in viability only in the SCADD and CPT2/MTPD patient cell lines under all 4 experimental conditions (Table S5). The antioxidants significantly improved viability not only in the SCADD and CPT2/MTPD cells, but also in the MCADD cells at 37°C (Table S6). NAC however, was significantly effective in improving viability in all patient cell lines under all 4 experimental conditions , as well as the normal controls under 3 of the experimental conditions at 0.5 mM, and in all 4 experimental conditions at 5.0 mM (Table S7). In a Bonferroni comparison, the effect of NAC 0.5 or 5 mM was significantly more effective in improving cellular viability than AO or Bezafibrate under all experimental conditions in the SCADD cell lines, p<0.001 (Tables S8, S9). This suggests that in SCADD cells, NAC has more extensive antioxidant actions than vitamin C and E or Bezafibrate. In the other cell lines, this difference in effect was less consistent (Tables S8, S9).

The mechanism for Bezafibrate reduction of oxidative stress is uncertain. Bezafibrate increases Complex I, III and IV enzyme activities in control cells and significantly increases activity of deficient RC complexes in certain RC deficient cells [36]. As EMA may interfere with Complexes I and III, leading to ROS generation, Bezafibrate may counteract, in part, these effects. Whether Bezafibrate restores short-chain FAO requires further studies.

The association between accumulated MCADD metabolites and oxidative stress has been investigated. In rat brain, octanoic and decanoic acids were shown to uncouple mitochondrial oxidative phosphorylation, provoke mitochondrial cytochrome c release, increase lipid and protein oxidation damage and decrease GSH levels [37], [38]. Our small number of MCADD patient fibroblasts survived significantly longer compared to 625G/G and 625G/A controls only at 37°C and in glucose-containing medium, p<0.05 (Table S3). With the addition of high temperature and/or glucose deprivation however, the MCADD cells were as sensitive to menadione-induced toxicity as these control cells, p>0.05. Furthermore, the MCADD fibroblasts were not significantly different to the 625A/A controls under all four experimental conditions. Therefore the MCADD fibroblasts did not appear to have a similar degree of risk for increased vulnerability to oxidative stress as seen in the SCADD and TFP/LCHADD fibroblasts.

Long-chain defect fibroblasts were more vulnerable to oxidative stress, comparable to the SCADD patient fibroblasts, p>0.05 (Fig. 1, Table S3). This may be attributable to the accumulation of palmitoyl-CoA and palmitoylcarnitine shown to have detergent properties on isolated canine myocytic sarcolemmal membranes and to potentiate ROS-induced lipid membrane peroxidative injury in ischemia [39]. Further, long-chain acyl-CoA's are potent inhibitors of mitochondrial adenine nucleotide transporter (ANT1) [40], which catalyzes exchange of ADP and ATP across the inner mitochondrial membrane [41] and is the overall rate-limiting step in oxidative phosphorylation [42]. Inhibition of ANT1 would lead to increased ROS.

In conclusion, there appear to be multifactorial mechanisms involved in the pathophysiology of clinical disease in SCAD deficiency including intrinsic factors which predispose to vulnerability to oxidative stress and protein unfolding, as well as exogenous stressors such as elevated temperature and hypoglycemia. Certain of these factors may also contribute to the pathophysiology of long-chain FAODs. The reversal of cellular toxicity *in vitro* with antioxidants and bezafibrate supports the role of these agents in maintaining mitochondrial homeostasis. We advocate rigorous management of fever in SCADD patients, avoidance of adverse conditions such as fasting, and prompt rescue during a catabolic crisis. Antioxidants and Bezafibrate may prove to be useful therapeutic agents for the prevention and amelioration of the neurological morbidity seen in SCAD deficiency.

## Supporting Information

Table S1 **Summary of short-chain acyl-CoA dehydrogenase deficiency (SCADD), medium-chain acyl-CoA dehydrogenase deficiency (MCADD), carnitine palmitoyltransferase 2 deficiency (CPT2D) and mitochondrial trifunctional protein deficiency (MTPD) patient and control data under the 4 experimental conditions.**
(PPT)

Table S2 **Summary of subgroup analysis with Bonferroni's Multiple Comparison test of short-chain acyl-CoA dehydrogenase deficiency (SCADD) under each experimental condition.**
(PPT)

Table S3 **Summary of subgroup analysis with Bonferroni's Multiple Comparison test under each experimental condition.**
(PPT)

Table S4 **Summary of menadione toxicity in the 4 SCADD patient genotypes.**
(PPT)

Table S5 **Effect of Bezafibrate intervention (200 and 400 µmol/L in SCADD fibroblasts only) on menadione toxicity in each FAO disorder under variable conditions.**
(PPT)

Table S6 **Effect of Antioxidant intervention on menadione toxicity in each FAO disorder under variable conditions.**
(PPT)

Table S7 **Effect of N-acetyl-cysteine (NAC, 0.5 and 5 mmol/L) intervention on menadione toxicity in each FAO disorder under variable conditions.**
(PPT)

Table S8 **Effect of N-acetyl-cysteine (NAC, 0.5 and 5 mmol/L) intervention on menadione toxicity in each FAO disorder and control lines under variable conditions, compared to AO.**
(PPT)

Table S9 **Effect of N-acetyl-cysteine (NAC, 0.5 and 5 mmol/L) intervention on menadione toxicity in each FAO disorder and control lines under variable conditions, compared to Bezafibrate.**
(PPT)

Figure S1 **Menadione toxicity assay with rescue in SCADD.** Rescue of SCADD (n = 11) from hypoglycemia and hyperthermia with glucose administration and normothermia, respectively, compared to SCADD cells with menadione toxicity at physiological conditions. Paired t-tests indicated by errors bars and p-values. This figure shows that rescue with glucose administration increased survival by 1.5×, compared to 3.1× increase in survival of SCADD cells rescued with normothermia. At 39°C, rescue with glucose and normothermia increased survival by 2.9×, compared to glucose rescue alone, 1.3×.
(TIF)
