# Supplementary material for: Vulnerability to Oxidative Stress In Vitro in Pathophysiology of Mitochondrial Short-Chain Acyl-CoA Dehydrogenase Deficiency: Response to Antioxidants
Source: PLoS One. 2011 Apr 1;6(4):e17534. doi: 10.1371/journal.pone.0017534 (PMC3069965; doi:10.1371/journal.pone.0017534)
Supplement: Table S1 — Summary of short-chain acyl-CoA dehydrogenase deficiency (SCADD), medium-chain acyl-CoA dehydrogenase deficiency (MCADD), carnitine palmitoyltransferase 2 deficiency (CPT2D) and mitochondrial trifunctional protein deficiency (MTPD) patient and control data under the 4 experimental conditions. (PPT) [file pone.0017534.s001.ppt]

## Slide 1
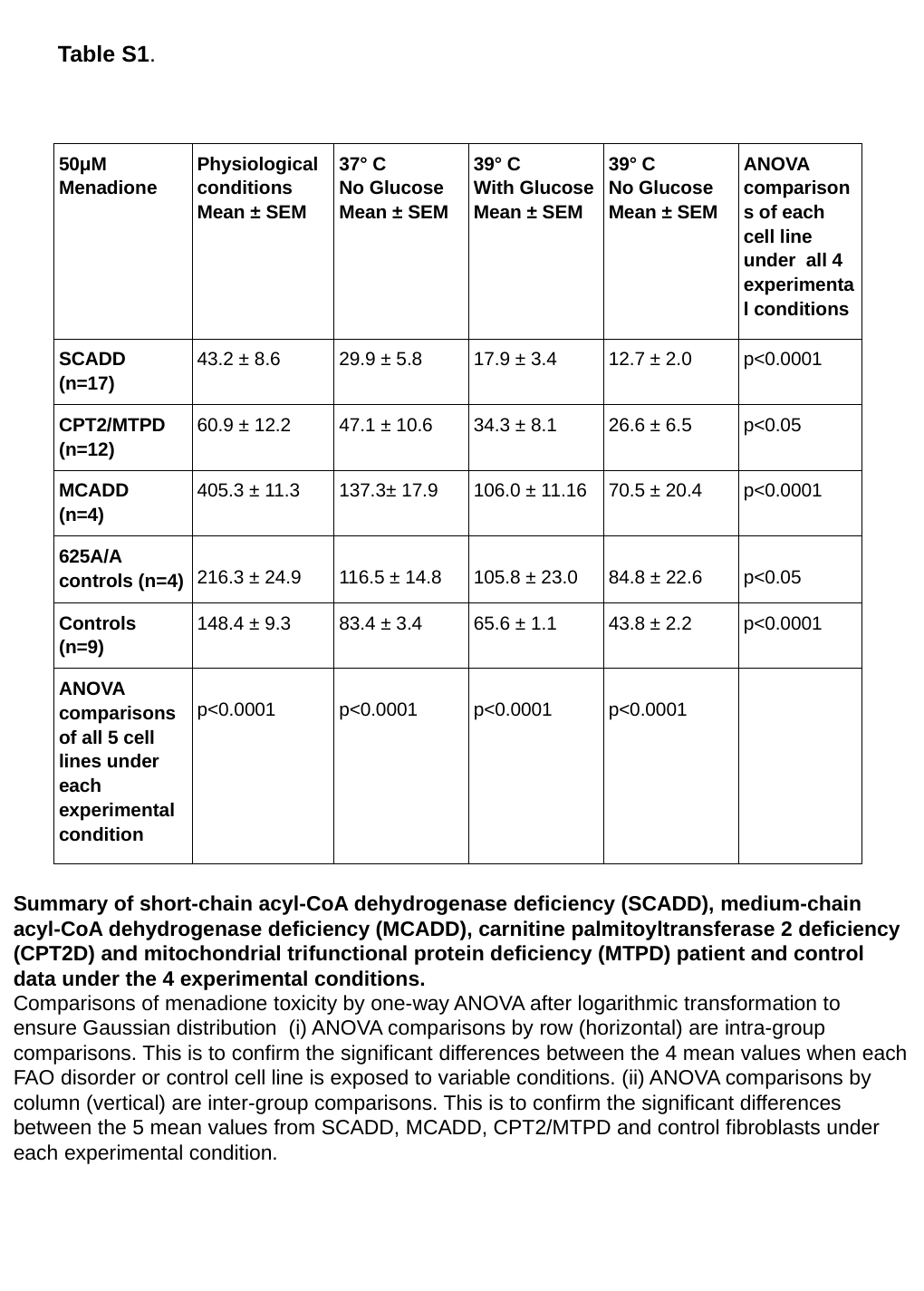

Table S1.
| 50μM Menadione | Physiological conditions Mean ± SEM | 37° C No Glucose Mean ± SEM | 39° C With Glucose Mean ± SEM | 39° C No Glucose Mean ± SEM | ANOVA comparisons of each cell line under all 4 experimental conditions |
| --- | --- | --- | --- | --- | --- |
| SCADD (n=17) | 43.2 ± 8.6 | 29.9 ± 5.8 | 17.9 ± 3.4 | 12.7 ± 2.0 | p<0.0001 |
| CPT2/MTPD (n=12) | 60.9 ± 12.2 | 47.1 ± 10.6 | 34.3 ± 8.1 | 26.6 ± 6.5 | p<0.05 |
| MCADD (n=4) | 405.3 ± 11.3 | 137.3± 17.9 | 106.0 ± 11.16 | 70.5 ± 20.4 | p<0.0001 |
| 625A/A controls (n=4) | 216.3 ± 24.9 | 116.5 ± 14.8 | 105.8 ± 23.0 | 84.8 ± 22.6 | p<0.05 |
| Controls (n=9) | 148.4 ± 9.3 | 83.4 ± 3.4 | 65.6 ± 1.1 | 43.8 ± 2.2 | p<0.0001 |
| ANOVA comparisons of all 5 cell lines under each experimental condition | p<0.0001 | p<0.0001 | p<0.0001 | p<0.0001 | |
Summary of short-chain acyl-CoA dehydrogenase deficiency (SCADD), medium-chain acyl-CoA dehydrogenase deficiency (MCADD), carnitine palmitoyltransferase 2 deficiency (CPT2D) and mitochondrial trifunctional protein deficiency (MTPD) patient and control data under the 4 experimental conditions.
Comparisons of menadione toxicity by one-way ANOVA after logarithmic transformation to ensure Gaussian distribution (i) ANOVA comparisons by row (horizontal) are intra-group comparisons. This is to confirm the significant differences between the 4 mean values when each FAO disorder or control cell line is exposed to variable conditions. (ii) ANOVA comparisons by column (vertical) are inter-group comparisons. This is to confirm the significant differences between the 5 mean values from SCADD, MCADD, CPT2/MTPD and control fibroblasts under each experimental condition.
