# Supplementary material for: Vulnerability to Oxidative Stress In Vitro in Pathophysiology of Mitochondrial Short-Chain Acyl-CoA Dehydrogenase Deficiency: Response to Antioxidants
Source: PLoS One. 2011 Apr 1;6(4):e17534. doi: 10.1371/journal.pone.0017534 (PMC3069965; doi:10.1371/journal.pone.0017534)
Supplement: Table S2 — Summary of subgroup analysis with Bonferroni's Multiple Comparison test of short-chain acyl-CoA dehydrogenase deficiency (SCADD) under each experimental condition. (PPT) [file pone.0017534.s002.ppt]

## Slide 1
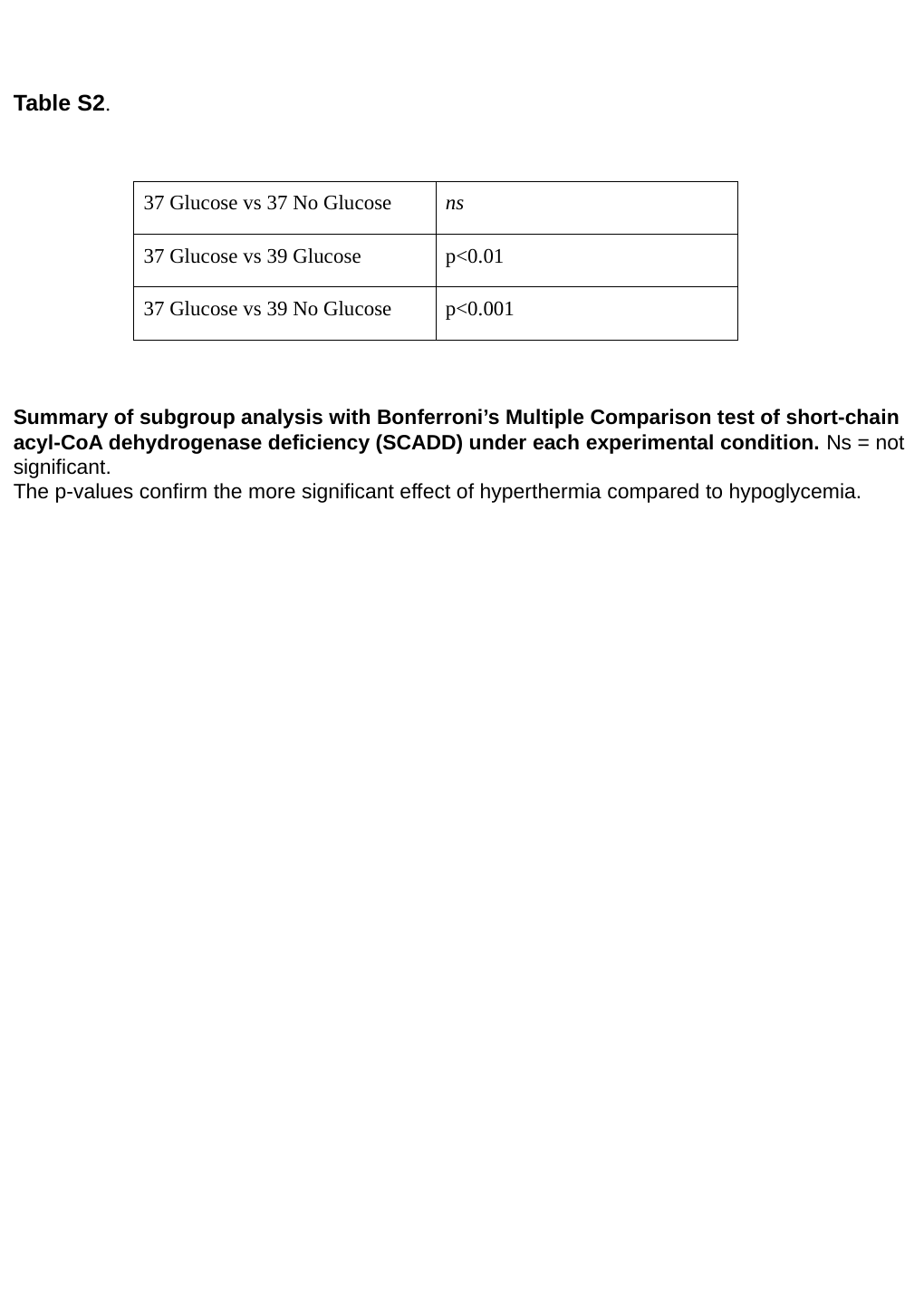

Table S2.
| 37 Glucose vs 37 No Glucose | ns |
| --- | --- |
| 37 Glucose vs 39 Glucose | p<0.01 |
| 37 Glucose vs 39 No Glucose | p<0.001 |
Summary of subgroup analysis with Bonferroni’s Multiple Comparison test of short-chain acyl-CoA dehydrogenase deficiency (SCADD) under each experimental condition. Ns = not significant.
The p-values confirm the more significant effect of hyperthermia compared to hypoglycemia.
