# Supplementary material for: Vulnerability to Oxidative Stress In Vitro in Pathophysiology of Mitochondrial Short-Chain Acyl-CoA Dehydrogenase Deficiency: Response to Antioxidants
Source: PLoS One. 2011 Apr 1;6(4):e17534. doi: 10.1371/journal.pone.0017534 (PMC3069965; doi:10.1371/journal.pone.0017534)
Supplement: Table S3 — Summary of subgroup analysis with Bonferroni's Multiple Comparison test under each experimental condition. (PPT) [file pone.0017534.s003.ppt]

## Slide 1
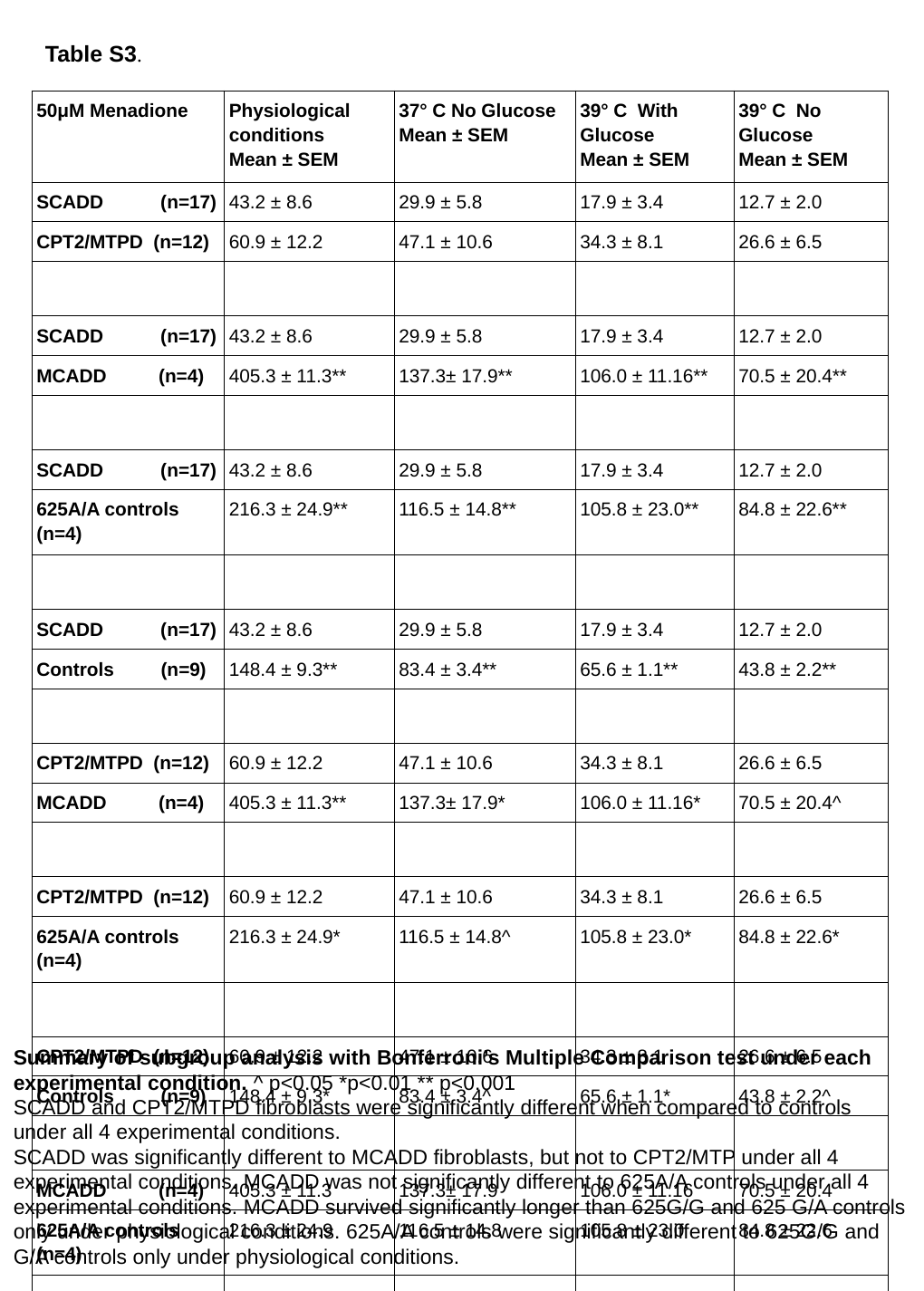

Table S3.
| 50μM Menadione | Physiological conditions Mean ± SEM | 37° C No Glucose Mean ± SEM | 39° C With Glucose Mean ± SEM | 39° C No Glucose Mean ± SEM |
| --- | --- | --- | --- | --- |
| SCADD (n=17) | 43.2 ± 8.6 | 29.9 ± 5.8 | 17.9 ± 3.4 | 12.7 ± 2.0 |
| CPT2/MTPD (n=12) | 60.9 ± 12.2 | 47.1 ± 10.6 | 34.3 ± 8.1 | 26.6 ± 6.5 |
| | | | | |
| SCADD (n=17) | 43.2 ± 8.6 | 29.9 ± 5.8 | 17.9 ± 3.4 | 12.7 ± 2.0 |
| MCADD (n=4) | 405.3 ± 11.3\*\* | 137.3± 17.9\*\* | 106.0 ± 11.16\*\* | 70.5 ± 20.4\*\* |
| | | | | |
| SCADD (n=17) | 43.2 ± 8.6 | 29.9 ± 5.8 | 17.9 ± 3.4 | 12.7 ± 2.0 |
| 625A/A controls (n=4) | 216.3 ± 24.9\*\* | 116.5 ± 14.8\*\* | 105.8 ± 23.0\*\* | 84.8 ± 22.6\*\* |
| | | | | |
| SCADD (n=17) | 43.2 ± 8.6 | 29.9 ± 5.8 | 17.9 ± 3.4 | 12.7 ± 2.0 |
| Controls (n=9) | 148.4 ± 9.3\*\* | 83.4 ± 3.4\*\* | 65.6 ± 1.1\*\* | 43.8 ± 2.2\*\* |
| | | | | |
| CPT2/MTPD (n=12) | 60.9 ± 12.2 | 47.1 ± 10.6 | 34.3 ± 8.1 | 26.6 ± 6.5 |
| MCADD (n=4) | 405.3 ± 11.3\*\* | 137.3± 17.9\* | 106.0 ± 11.16\* | 70.5 ± 20.4^ |
| | | | | |
| CPT2/MTPD (n=12) | 60.9 ± 12.2 | 47.1 ± 10.6 | 34.3 ± 8.1 | 26.6 ± 6.5 |
| 625A/A controls (n=4) | 216.3 ± 24.9\* | 116.5 ± 14.8^ | 105.8 ± 23.0\* | 84.8 ± 22.6\* |
| | | | | |
| CPT2/MTPD (n=12) | 60.9 ± 12.2 | 47.1 ± 10.6 | 34.3 ± 8.1 | 26.6 ± 6.5 |
| Controls (n=9) | 148.4 ± 9.3\* | 83.4 ± 3.4^ | 65.6 ± 1.1\* | 43.8 ± 2.2^ |
| | | | | |
| MCADD (n=4) | 405.3 ± 11.3 | 137.3± 17.9 | 106.0 ± 11.16 | 70.5 ± 20.4 |
| 625A/A controls (n=4) | 216.3 ± 24.9 | 116.5 ± 14.8 | 105.8 ± 23.0 | 84.8 ± 22.6 |
| | | | | |
| MCADD (n=4) | 405.3 ± 11.3 | 137.3± 17.9 | 106.0 ± 11.16 | 70.5 ± 20.4 |
| Controls (n=9) | 148.4 ± 9.3^ | 83.4 ± 3.4 | 65.6 ± 1.1 | 43.8 ± 2.2 |
| | | | | |
| 625A/A controls (n=4) | 216.3 ± 24.9 | 116.5 ± 14.8 | 105.8 ± 23.0 | 84.8 ± 22.6 |
| Controls (n=9) | 148.4 ± 9.3^ | 83.4 ± 3.4 | 65.6 ± 1.1 | 43.8 ± 2.2 |
Summary of subgroup analysis with Bonferroni’s Multiple Comparison test under each experimental condition. ^ p<0.05 *p<0.01 ** p<0.001
SCADD and CPT2/MTPD fibroblasts were significantly different when compared to controls under all 4 experimental conditions.
SCADD was significantly different to MCADD fibroblasts, but not to CPT2/MTP under all 4 experimental conditions. MCADD was not significantly different to 625A/A controls under all 4 experimental conditions. MCADD survived significantly longer than 625G/G and 625 G/A controls only under physiological conditions. 625A/A controls were significantly different to 625G/G and G/A controls only under physiological conditions.
