# Supplementary material for: Vulnerability to Oxidative Stress In Vitro in Pathophysiology of Mitochondrial Short-Chain Acyl-CoA Dehydrogenase Deficiency: Response to Antioxidants
Source: PLoS One. 2011 Apr 1;6(4):e17534. doi: 10.1371/journal.pone.0017534 (PMC3069965; doi:10.1371/journal.pone.0017534)
Supplement: Table S4 — Summary of menadione toxicity in the 4 SCADD patient genotypes. (PPT) [file pone.0017534.s004.ppt]

## Slide 1
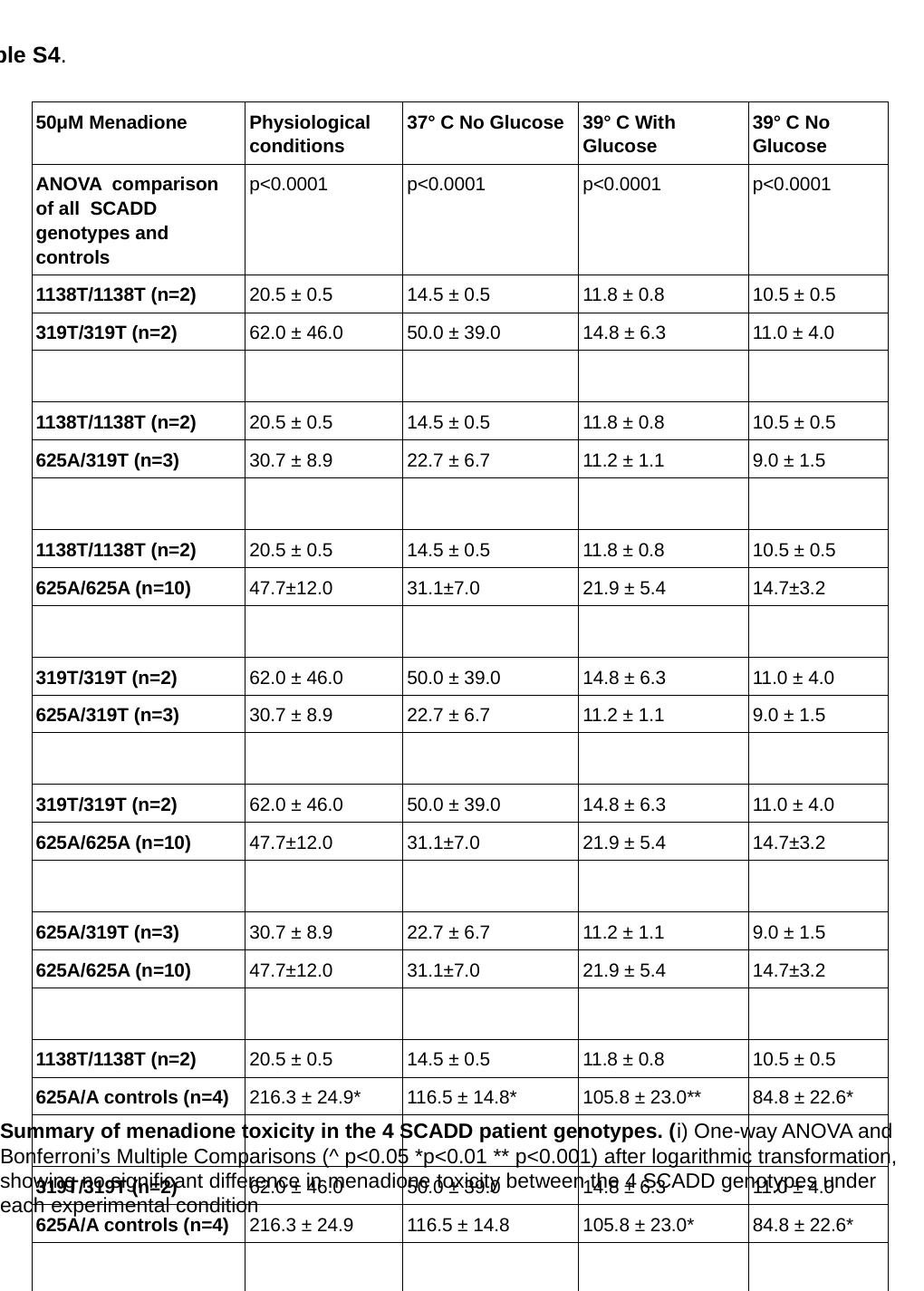

Table S4.
| 50μM Menadione | Physiological conditions | 37° C No Glucose | 39° C With Glucose | 39° C No Glucose |
| --- | --- | --- | --- | --- |
| ANOVA comparison of all SCADD genotypes and controls | p<0.0001 | p<0.0001 | p<0.0001 | p<0.0001 |
| 1138T/1138T (n=2) | 20.5 ± 0.5 | 14.5 ± 0.5 | 11.8 ± 0.8 | 10.5 ± 0.5 |
| 319T/319T (n=2) | 62.0 ± 46.0 | 50.0 ± 39.0 | 14.8 ± 6.3 | 11.0 ± 4.0 |
| | | | | |
| 1138T/1138T (n=2) | 20.5 ± 0.5 | 14.5 ± 0.5 | 11.8 ± 0.8 | 10.5 ± 0.5 |
| 625A/319T (n=3) | 30.7 ± 8.9 | 22.7 ± 6.7 | 11.2 ± 1.1 | 9.0 ± 1.5 |
| | | | | |
| 1138T/1138T (n=2) | 20.5 ± 0.5 | 14.5 ± 0.5 | 11.8 ± 0.8 | 10.5 ± 0.5 |
| 625A/625A (n=10) | 47.7±12.0 | 31.1±7.0 | 21.9 ± 5.4 | 14.7±3.2 |
| | | | | |
| 319T/319T (n=2) | 62.0 ± 46.0 | 50.0 ± 39.0 | 14.8 ± 6.3 | 11.0 ± 4.0 |
| 625A/319T (n=3) | 30.7 ± 8.9 | 22.7 ± 6.7 | 11.2 ± 1.1 | 9.0 ± 1.5 |
| | | | | |
| 319T/319T (n=2) | 62.0 ± 46.0 | 50.0 ± 39.0 | 14.8 ± 6.3 | 11.0 ± 4.0 |
| 625A/625A (n=10) | 47.7±12.0 | 31.1±7.0 | 21.9 ± 5.4 | 14.7±3.2 |
| | | | | |
| 625A/319T (n=3) | 30.7 ± 8.9 | 22.7 ± 6.7 | 11.2 ± 1.1 | 9.0 ± 1.5 |
| 625A/625A (n=10) | 47.7±12.0 | 31.1±7.0 | 21.9 ± 5.4 | 14.7±3.2 |
| | | | | |
| 1138T/1138T (n=2) | 20.5 ± 0.5 | 14.5 ± 0.5 | 11.8 ± 0.8 | 10.5 ± 0.5 |
| 625A/A controls (n=4) | 216.3 ± 24.9\* | 116.5 ± 14.8\* | 105.8 ± 23.0\*\* | 84.8 ± 22.6\* |
| | | | | |
| 319T/319T (n=2) | 62.0 ± 46.0 | 50.0 ± 39.0 | 14.8 ± 6.3 | 11.0 ± 4.0 |
| 625A/A controls (n=4) | 216.3 ± 24.9 | 116.5 ± 14.8 | 105.8 ± 23.0\* | 84.8 ± 22.6\* |
| | | | | |
| 625A/319T (n=3) | 30.7 ± 8.9 | 22.7 ± 6.7 | 11.2 ± 1.1 | 9.0 ± 1.5 |
| 625A/A controls (n=4) | 216.3 ± 24.9\* | 116.5 ± 14.8^ | 105.8 ± 23.0\*\* | 84.8 ± 22.6\*\* |
| | | | | |
| 625A/625A (n=10) | 47.7±12.0 | 31.1±7.0 | 21.9 ± 5.4 | 14.7±3.2 |
| 625A/A controls (n=4) | 216.3 ± 24.9\*\* | 116.5 ± 14.8\* | 105.8 ± 23.0\*\* | 84.8 ± 22.6\*\* |
| | | | | |
| 625A/625A (n=10) | 47.7±12.0 | 31.1±7.0 | 21.9 ± 5.4 | 14.7±3.2 |
| Controls (n=9) | 148.4 ± 9.3\*\* | 83.4 ± 3.4\* | 65.6 ± 1.1\*\* | 43.8 ± 2.2\*\* |
Summary of menadione toxicity in the 4 SCADD patient genotypes. (i) One-way ANOVA and Bonferroni’s Multiple Comparisons (^ p<0.05 *p<0.01 ** p<0.001) after logarithmic transformation, showing no significant difference in menadione toxicity between the 4 SCADD genotypes under each experimental condition
