# Supplementary material for: Vulnerability to Oxidative Stress In Vitro in Pathophysiology of Mitochondrial Short-Chain Acyl-CoA Dehydrogenase Deficiency: Response to Antioxidants
Source: PLoS One. 2011 Apr 1;6(4):e17534. doi: 10.1371/journal.pone.0017534 (PMC3069965; doi:10.1371/journal.pone.0017534)
Supplement: Table S5 — Effect of Bezafibrate intervention (200 and 400 µmol/L in SCADD fibroblasts only) on menadione toxicity in each FAO disorder under variable conditions. (PPT) [file pone.0017534.s005.ppt]

## Slide 1
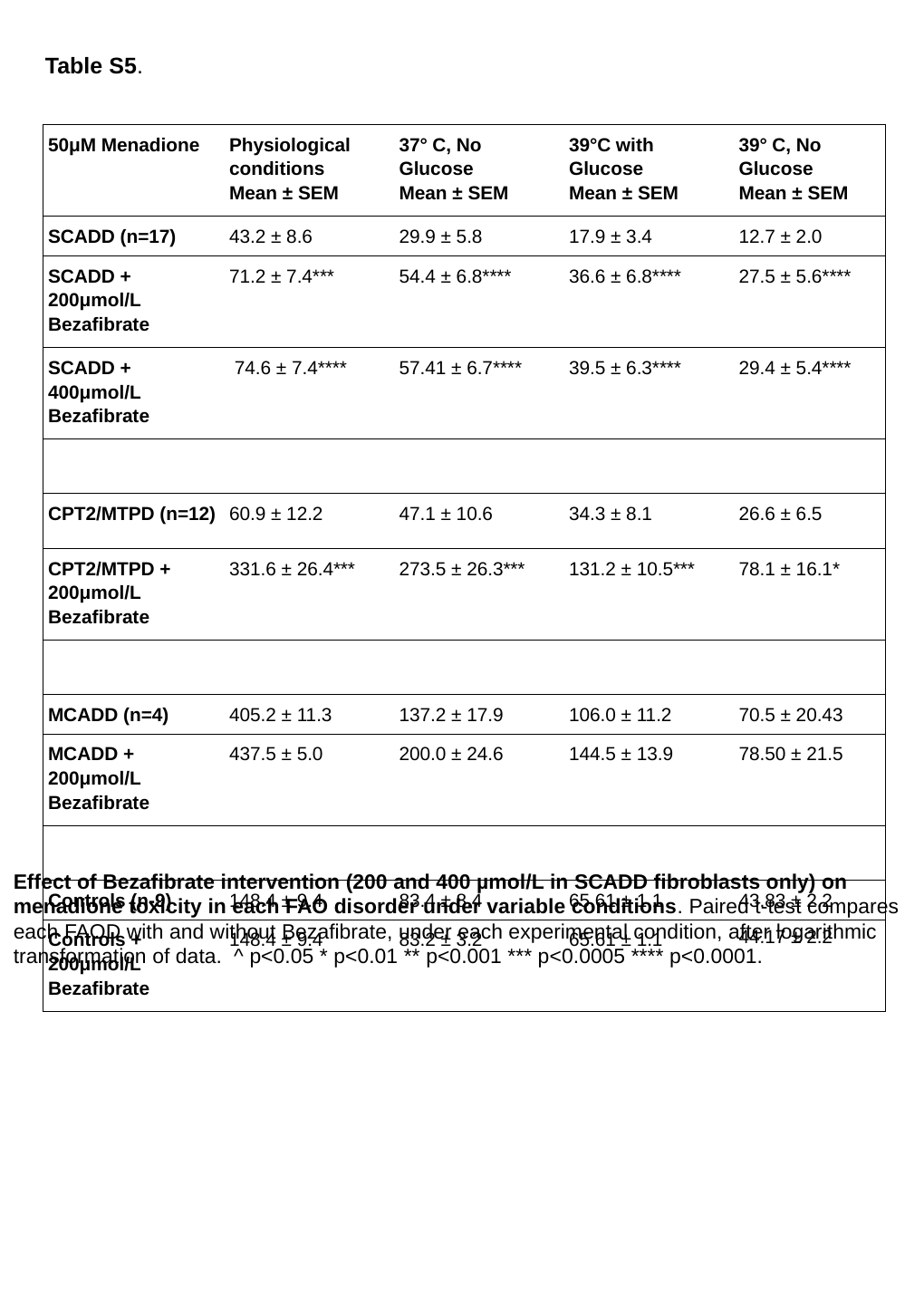

Table S5.
| 50μM Menadione | Physiological conditions Mean ± SEM | 37° C, No Glucose Mean ± SEM | 39°C with Glucose Mean ± SEM | 39° C, No Glucose Mean ± SEM |
| --- | --- | --- | --- | --- |
| SCADD (n=17) | 43.2 ± 8.6 | 29.9 ± 5.8 | 17.9 ± 3.4 | 12.7 ± 2.0 |
| SCADD + 200μmol/L Bezafibrate | 71.2 ± 7.4\*\*\* | 54.4 ± 6.8\*\*\*\* | 36.6 ± 6.8\*\*\*\* | 27.5 ± 5.6\*\*\*\* |
| SCADD + 400μmol/L Bezafibrate | 74.6 ± 7.4\*\*\*\* | 57.41 ± 6.7\*\*\*\* | 39.5 ± 6.3\*\*\*\* | 29.4 ± 5.4\*\*\*\* |
| | | | | |
| CPT2/MTPD (n=12) | 60.9 ± 12.2 | 47.1 ± 10.6 | 34.3 ± 8.1 | 26.6 ± 6.5 |
| CPT2/MTPD + 200μmol/L Bezafibrate | 331.6 ± 26.4\*\*\* | 273.5 ± 26.3\*\*\* | 131.2 ± 10.5\*\*\* | 78.1 ± 16.1\* |
| | | | | |
| MCADD (n=4) | 405.2 ± 11.3 | 137.2 ± 17.9 | 106.0 ± 11.2 | 70.5 ± 20.43 |
| MCADD + 200μmol/L Bezafibrate | 437.5 ± 5.0 | 200.0 ± 24.6 | 144.5 ± 13.9 | 78.50 ± 21.5 |
| | | | | |
| Controls (n-9) | 148.4 ± 9.4 | 83.4 ± 8.4 | 65.61 ± 1.1 | 43.83 ± 2.2 |
| Controls + 200μmol/L Bezafibrate | 148.4 ± 9.4 | 83.2 ± 3.2 | 65.61 ± 1.1 | 44.17 ± 2.2 |
Effect of Bezafibrate intervention (200 and 400 µmol/L in SCADD fibroblasts only) on menadione toxicity in each FAO disorder under variable conditions. Paired t-test compares each FAOD with and without Bezafibrate, under each experimental condition, after logarithmic transformation of data. ^ p<0.05 * p<0.01 ** p<0.001 *** p<0.0005 **** p<0.0001.
