# Supplementary material for: Vulnerability to Oxidative Stress In Vitro in Pathophysiology of Mitochondrial Short-Chain Acyl-CoA Dehydrogenase Deficiency: Response to Antioxidants
Source: PLoS One. 2011 Apr 1;6(4):e17534. doi: 10.1371/journal.pone.0017534 (PMC3069965; doi:10.1371/journal.pone.0017534)
Supplement: Table S6 — Effect of Antioxidant intervention on menadione toxicity in each FAO disorder under variable conditions. (PPT) [file pone.0017534.s006.ppt]

## Slide 1
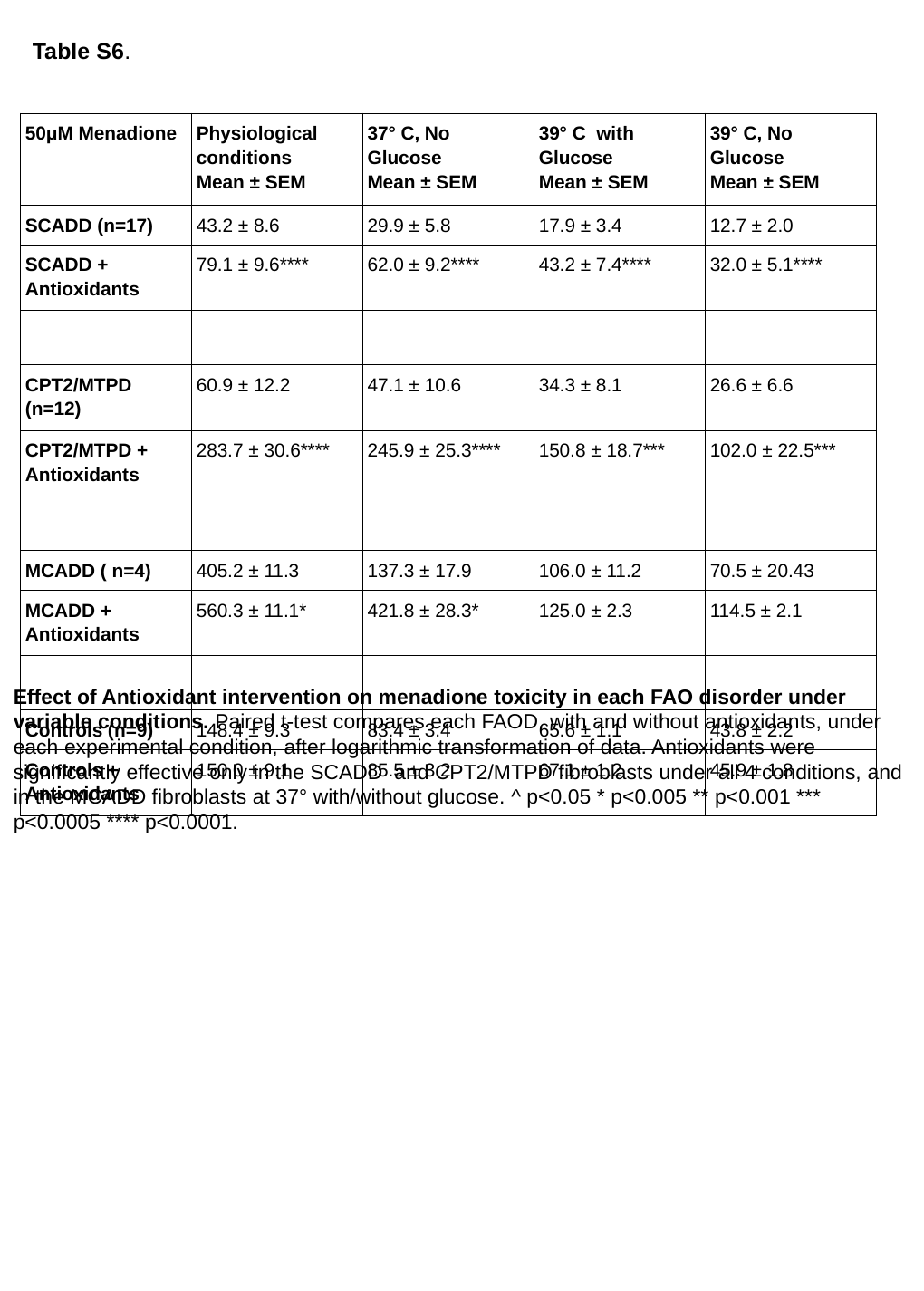

Table S6.
| 50μM Menadione | Physiological conditions Mean ± SEM | 37° C, No Glucose Mean ± SEM | 39° C with Glucose Mean ± SEM | 39° C, No Glucose Mean ± SEM |
| --- | --- | --- | --- | --- |
| SCADD (n=17) | 43.2 ± 8.6 | 29.9 ± 5.8 | 17.9 ± 3.4 | 12.7 ± 2.0 |
| SCADD + Antioxidants | 79.1 ± 9.6\*\*\*\* | 62.0 ± 9.2\*\*\*\* | 43.2 ± 7.4\*\*\*\* | 32.0 ± 5.1\*\*\*\* |
| | | | | |
| CPT2/MTPD (n=12) | 60.9 ± 12.2 | 47.1 ± 10.6 | 34.3 ± 8.1 | 26.6 ± 6.6 |
| CPT2/MTPD + Antioxidants | 283.7 ± 30.6\*\*\*\* | 245.9 ± 25.3\*\*\*\* | 150.8 ± 18.7\*\*\* | 102.0 ± 22.5\*\*\* |
| | | | | |
| MCADD ( n=4) | 405.2 ± 11.3 | 137.3 ± 17.9 | 106.0 ± 11.2 | 70.5 ± 20.43 |
| MCADD + Antioxidants | 560.3 ± 11.1\* | 421.8 ± 28.3\* | 125.0 ± 2.3 | 114.5 ± 2.1 |
| | | | | |
| Controls (n=9) | 148.4 ± 9.3 | 83.4 ± 3.4 | 65.6 ± 1.1 | 43.8 ± 2.2 |
| Controls + Antioxidants | 150.0 ± 9.1 | 85.5 ± 3.2 | 67.1 ± 1.2 | 45.9 ± 1.8 |
Effect of Antioxidant intervention on menadione toxicity in each FAO disorder under variable conditions. Paired t-test compares each FAOD, with and without antioxidants, under each experimental condition, after logarithmic transformation of data. Antioxidants were significantly effective only in the SCADD and CPT2/MTPD fibroblasts under all 4 conditions, and in the MCADD fibroblasts at 37° with/without glucose. ^ p<0.05 * p<0.005 ** p<0.001 *** p<0.0005 **** p<0.0001.
