# Supplementary material for: Vulnerability to Oxidative Stress In Vitro in Pathophysiology of Mitochondrial Short-Chain Acyl-CoA Dehydrogenase Deficiency: Response to Antioxidants
Source: PLoS One. 2011 Apr 1;6(4):e17534. doi: 10.1371/journal.pone.0017534 (PMC3069965; doi:10.1371/journal.pone.0017534)
Supplement: Table S7 — Effect of N-acetyl-cysteine (NAC, 0.5 and 5 mmol/L) intervention on menadione toxicity in each FAO disorder under variable conditions. (PPT) [file pone.0017534.s007.ppt]

## Slide 1
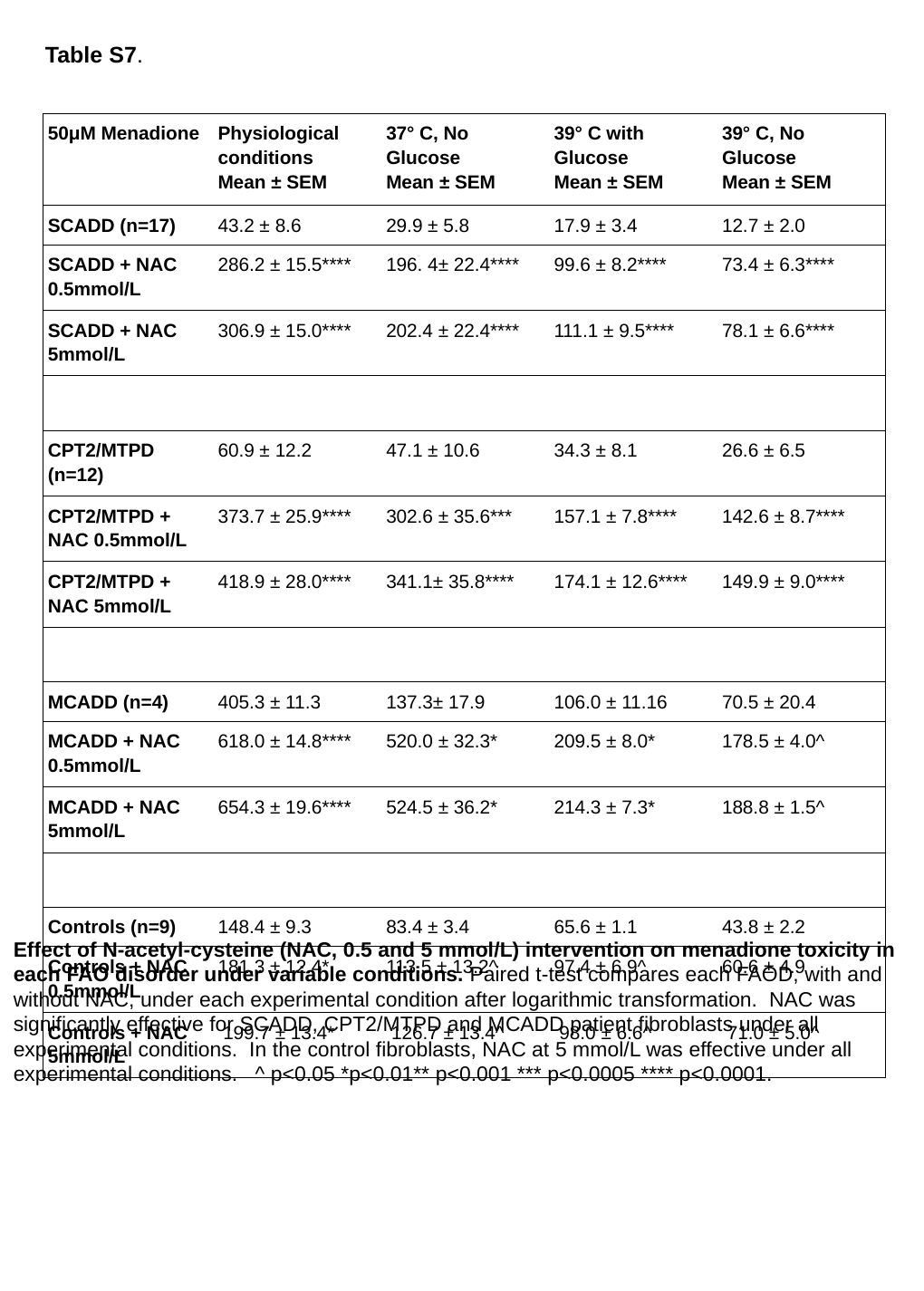

Table S7.
| 50μM Menadione | Physiological conditions Mean ± SEM | 37° C, No Glucose Mean ± SEM | 39° C with Glucose Mean ± SEM | 39° C, No Glucose Mean ± SEM |
| --- | --- | --- | --- | --- |
| SCADD (n=17) | 43.2 ± 8.6 | 29.9 ± 5.8 | 17.9 ± 3.4 | 12.7 ± 2.0 |
| SCADD + NAC 0.5mmol/L | 286.2 ± 15.5\*\*\*\* | 196. 4± 22.4\*\*\*\* | 99.6 ± 8.2\*\*\*\* | 73.4 ± 6.3\*\*\*\* |
| SCADD + NAC 5mmol/L | 306.9 ± 15.0\*\*\*\* | 202.4 ± 22.4\*\*\*\* | 111.1 ± 9.5\*\*\*\* | 78.1 ± 6.6\*\*\*\* |
| | | | | |
| CPT2/MTPD (n=12) | 60.9 ± 12.2 | 47.1 ± 10.6 | 34.3 ± 8.1 | 26.6 ± 6.5 |
| CPT2/MTPD + NAC 0.5mmol/L | 373.7 ± 25.9\*\*\*\* | 302.6 ± 35.6\*\*\* | 157.1 ± 7.8\*\*\*\* | 142.6 ± 8.7\*\*\*\* |
| CPT2/MTPD + NAC 5mmol/L | 418.9 ± 28.0\*\*\*\* | 341.1± 35.8\*\*\*\* | 174.1 ± 12.6\*\*\*\* | 149.9 ± 9.0\*\*\*\* |
| | | | | |
| MCADD (n=4) | 405.3 ± 11.3 | 137.3± 17.9 | 106.0 ± 11.16 | 70.5 ± 20.4 |
| MCADD + NAC 0.5mmol/L | 618.0 ± 14.8\*\*\*\* | 520.0 ± 32.3\* | 209.5 ± 8.0\* | 178.5 ± 4.0^ |
| MCADD + NAC 5mmol/L | 654.3 ± 19.6\*\*\*\* | 524.5 ± 36.2\* | 214.3 ± 7.3\* | 188.8 ± 1.5^ |
| | | | | |
| Controls (n=9) | 148.4 ± 9.3 | 83.4 ± 3.4 | 65.6 ± 1.1 | 43.8 ± 2.2 |
| Controls + NAC 0.5mmol/L | 181.3 ± 12.4\* | 113.5 ± 13.2^ | 97.4 ± 6.9^ | 60.6 ± 4.9 |
| Controls + NAC 5mmol/L | 199.7 ± 13.4\* | 126.7 ± 13.4^ | 98.0 ± 6.6^ | 71.0 ± 5.0^ |
Effect of N-acetyl-cysteine (NAC, 0.5 and 5 mmol/L) intervention on menadione toxicity in each FAO disorder under variable conditions. Paired t-test compares each FAOD, with and without NAC, under each experimental condition after logarithmic transformation. NAC was significantly effective for SCADD, CPT2/MTPD and MCADD patient fibroblasts under all experimental conditions. In the control fibroblasts, NAC at 5 mmol/L was effective under all experimental conditions. ^ p<0.05 *p<0.01** p<0.001 *** p<0.0005 **** p<0.0001.
