# Supplementary material for: Vulnerability to Oxidative Stress In Vitro in Pathophysiology of Mitochondrial Short-Chain Acyl-CoA Dehydrogenase Deficiency: Response to Antioxidants
Source: PLoS One. 2011 Apr 1;6(4):e17534. doi: 10.1371/journal.pone.0017534 (PMC3069965; doi:10.1371/journal.pone.0017534)
Supplement: Table S9 — Effect of N-acetyl-cysteine (NAC, 0.5 and 5 mmol/L) intervention on menadione toxicity in each FAO disorder and control lines under variable conditions, compared to Bezafibrate. (PPT) [file pone.0017534.s009.ppt]

## Slide 1
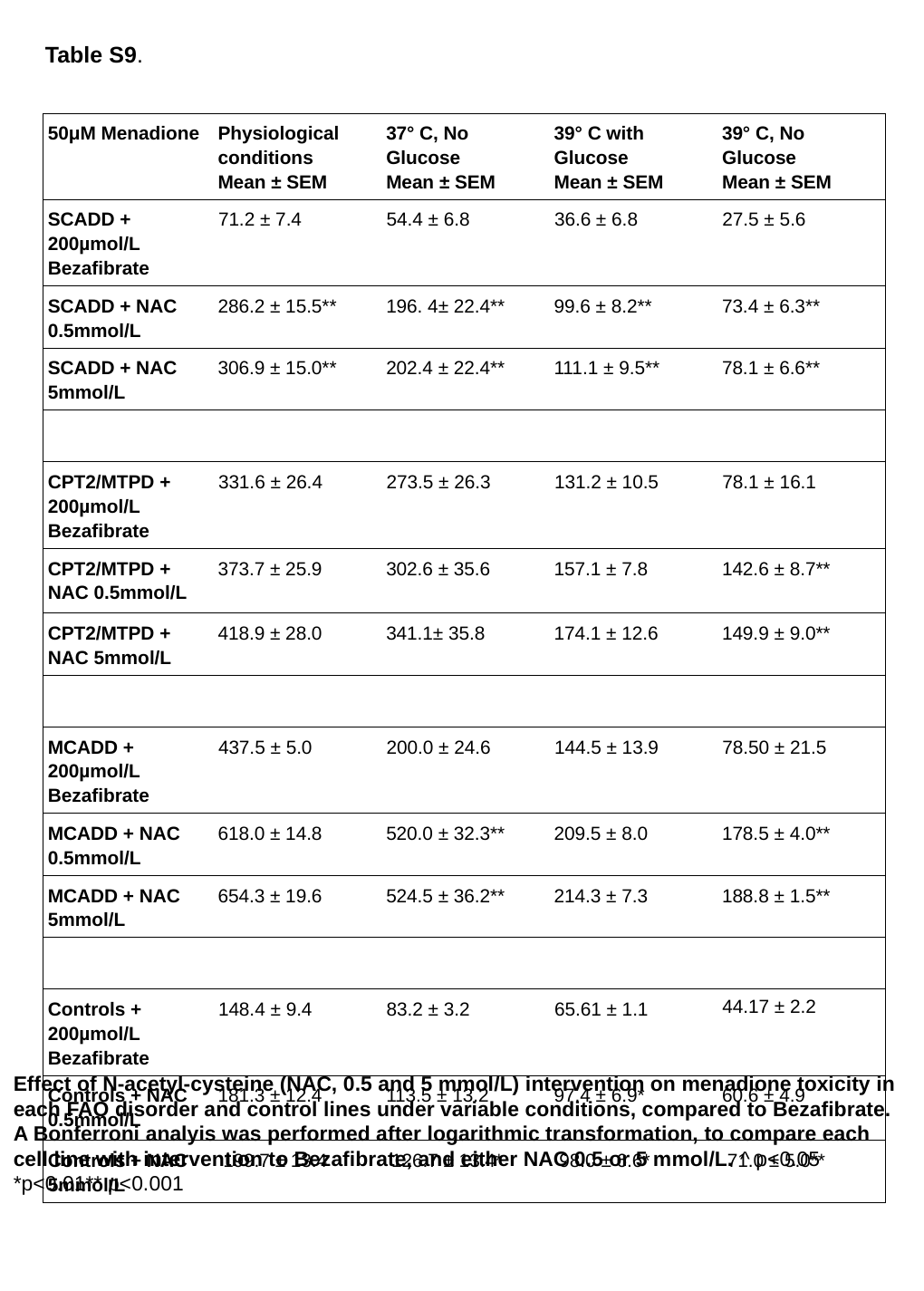

Table S9.
| 50μM Menadione | Physiological conditions Mean ± SEM | 37° C, No Glucose Mean ± SEM | 39° C with Glucose Mean ± SEM | 39° C, No Glucose Mean ± SEM |
| --- | --- | --- | --- | --- |
| SCADD + 200µmol/L Bezafibrate | 71.2 ± 7.4 | 54.4 ± 6.8 | 36.6 ± 6.8 | 27.5 ± 5.6 |
| SCADD + NAC 0.5mmol/L | 286.2 ± 15.5\*\* | 196. 4± 22.4\*\* | 99.6 ± 8.2\*\* | 73.4 ± 6.3\*\* |
| SCADD + NAC 5mmol/L | 306.9 ± 15.0\*\* | 202.4 ± 22.4\*\* | 111.1 ± 9.5\*\* | 78.1 ± 6.6\*\* |
| | | | | |
| CPT2/MTPD + 200µmol/L Bezafibrate | 331.6 ± 26.4 | 273.5 ± 26.3 | 131.2 ± 10.5 | 78.1 ± 16.1 |
| CPT2/MTPD + NAC 0.5mmol/L | 373.7 ± 25.9 | 302.6 ± 35.6 | 157.1 ± 7.8 | 142.6 ± 8.7\*\* |
| CPT2/MTPD + NAC 5mmol/L | 418.9 ± 28.0 | 341.1± 35.8 | 174.1 ± 12.6 | 149.9 ± 9.0\*\* |
| | | | | |
| MCADD + 200µmol/L Bezafibrate | 437.5 ± 5.0 | 200.0 ± 24.6 | 144.5 ± 13.9 | 78.50 ± 21.5 |
| MCADD + NAC 0.5mmol/L | 618.0 ± 14.8 | 520.0 ± 32.3\*\* | 209.5 ± 8.0 | 178.5 ± 4.0\*\* |
| MCADD + NAC 5mmol/L | 654.3 ± 19.6 | 524.5 ± 36.2\*\* | 214.3 ± 7.3 | 188.8 ± 1.5\*\* |
| | | | | |
| Controls + 200µmol/L Bezafibrate | 148.4 ± 9.4 | 83.2 ± 3.2 | 65.61 ± 1.1 | 44.17 ± 2.2 |
| Controls + NAC 0.5mmol/L | 181.3 ± 12.4 | 113.5 ± 13.2 | 97.4 ± 6.9\* | 60.6 ± 4.9 |
| Controls + NAC 5mmol/L | 199.7 ± 13.4 | 126.7 ± 13.4\* | 98.0 ± 6.6\* | 71.0 ± 5.0\*\* |
Effect of N-acetyl-cysteine (NAC, 0.5 and 5 mmol/L) intervention on menadione toxicity in each FAO disorder and control lines under variable conditions, compared to Bezafibrate. A Bonferroni analyis was performed after logarithmic transformation, to compare each cell line with intervention to Bezafibrate, and either NAC 0.5 or 5 mmol/L. ^ p<0.05 *p<0.01** p<0.001
